# Metagenomics of an Alkaline Hot Spring in Galicia (Spain): Microbial Diversity Analysis and Screening for Novel Lipolytic Enzymes

**DOI:** 10.3389/fmicb.2015.01291

**Published:** 2015-11-20

**Authors:** Olalla López-López, Kamila Knapik, Maria-Esperanza Cerdán, María-Isabel González-Siso

**Affiliations:** Grupo EXPRELA, Departamento de Bioloxía Celular e Molecular, Facultade de Ciencias, Centro de Investigacións Científicas Avanzadas, Universidade da CoruñaA Coruña, Spain

**Keywords:** metagenomics, esterase, beta-lactamase, alkaline hot spring, biodiversity, next-generation sequencing

## Abstract

A fosmid library was constructed with the metagenomic DNA from the water of the Lobios hot spring (76°C, pH = 8.2) located in Ourense (Spain). Metagenomic sequencing of the fosmid library allowed the assembly of 9722 contigs ranging in size from 500 to 56,677 bp and spanning ~18 Mbp. 23,207 ORFs (Open Reading Frames) were predicted from the assembly. Biodiversity was explored by taxonomic classification and it revealed that bacteria were predominant, while the archaea were less abundant. The six most abundant bacterial phyla were Deinococcus-Thermus, Proteobacteria, Firmicutes, Acidobacteria, Aquificae, and Chloroflexi. Within the archaeal superkingdom, the phylum Thaumarchaeota was predominant with the dominant species “*Candidatus Caldiarchaeum subterraneum*.” Functional classification revealed the genes associated to one-carbon metabolism as the most abundant. Both taxonomic and functional classifications showed a mixture of different microbial metabolic patterns: aerobic and anaerobic, chemoorganotrophic and chemolithotrophic, autotrophic and heterotrophic. Remarkably, the presence of genes encoding enzymes with potential biotechnological interest, such as xylanases, galactosidases, proteases, and lipases, was also revealed in the metagenomic library. Functional screening of this library was subsequently done looking for genes encoding lipolytic enzymes. Six genes conferring lipolytic activity were identified and one was cloned and characterized. This gene was named *LOB4Est* and it was expressed in a yeast mesophilic host. *LOB4Est* codes for a novel esterase of family VIII, with sequence similarity to β-lactamases, but with unusual wide substrate specificity. When the enzyme was purified from the mesophilic host it showed half-life of 1 h and 43 min at 50°C, and maximal activity at 40°C and pH 7.5 with *p*-nitrophenyl-laurate as substrate. Interestingly, the enzyme retained more than 80% of maximal activity in a broad range of pH from 6.5 to 8.

## Introduction

Thermophilic microorganisms, which live in environments with extremely high temperature, produce thermostable enzymes that show numerous industrial applications; some of these enzymes are stable and active at temperatures even higher than the optimal for the growth of the producing microorganisms (Haki and Rakshit, 2003). The availability of the so called thermozymes is opening up new horizons in a variety of biocatalyzed processes that are performed in harsh conditions.

Lipases and esterases are enzymes of great interest for industrial and biotechnological purposes. Food processing, cosmetic industry, fine chemical synthesis, waste treatment or laundry industry are only a few examples (Bornscheuer, 2002; Jaeger and Eggert, 2002; Haki and Rakshit, 2003; Joseph et al., 2007). Most processes where lipases and esterases are employed require reaction temperatures above 45°C (Haki and Rakshit, 2003). The search of thermostable lipolytic enzymes, with activity toward so far untreatable substrates, is crucial to improve the industrial biotransformations in which these enzymes are currently used, but also to develop new applications in other fields.

Metagenomics provides a powerful tool to explore the biodiversity of catalysis in nature, since it is known that enzymes from microorganisms that have not efficient conditions of culture in laboratory (99% of total; Amann et al., 1995) cannot be identified through other methods. Hot springs are natural habitats of thermophilic microorganisms, producing thermostable enzymes with catalytic activity at high temperatures. These features, in addition to the intrinsic resistance of thermophilic lipases and esterases to the presence of organic solvents or extreme pH values (Bornscheuer, 2002) highlight them as robust and versatile biocatalysts for industrial applications.

Galician region, in the North-West of Spain, harbors a great number of springs of mineral water of meteoric origin. Rainwater descends through fractures into the subsoil, and circulates changing its chemical composition and temperature before it emerges in natural springs. Many of these springs, located in big and deep fractures, heat the circulating water to high temperatures (Ramírez Ortega et al., 2007). Several thermophilic microorganims have been isolated from these Galician hot-springs, cultured in laboratory and used to produce lipolytic enzymes that have been characterized (Deive et al., 2013). However, microbial biodiversity and metagenomic potential, as source of lipolytic enzymes, of these valuable hot-springs have never been studied to date.

For this work, we chose the Lobios (Ourense) hot spring due to its high temperature (76°C) and alkaline pH (8.2). Such an environment can be a source of novel thermostable and alkaline-tolerant enzymes with industrial value. We generated a metagenomic library from the thermal water of this hot spring, using the fosmid pCC1FOS. The constructed library was sequenced and reads were assembled and analyzed to draw a picture of the microbial diversity captured into the Lobios library. The metagenomic library was also subjected to functional screening for lipolytic activity using tributyrin agar plates. One positive clone was selected for cloning, expression and characterization of the recombinant enzyme using the yeast *Saccharomyces cerevisiae* BJ3505 as heterologous host and the plasmid YEpFLAG-1 as expression vector. This enzyme has remarkable characteristics because it is stable at high temperature and also combines wide substrate specificity and operational pH range.

## Materials and methods

### Sampling

Thermal water was collected from Lobios hot spring (GPS 41.86113, −8.1062), in Ourense (Galician region, Spain). Twenty five Liter of groundwater sample was collected into a prewashed with 70% ethanol and rinsed with thermal water bottle. Sampling was performed directly from a borehole and not from a pool or reservoir exposed to light. Water sample (temperature >76°C and pH > 8.2) was stored at room temperature and processed the next day when it was filtered through a nitrocellulose filter of 0.2 μm cut-off, using a bottle top filter holder (Nalgene) and a vacuum pump. Filter with trapped microorganisms was stored at 4°C until metagenomic DNA extraction.

### Construction of metagenomic library

Total DNA was isolated from the filter using the Metagenomic DNA Isolation Kit for Water (Epicentre Biotechnologies). The high molecular weight DNA obtained was used directly to construct a metagenomic fosmid library with the fosmid pCC1FOS, using the Copy Control Fosmid Library Production kit (Epicentre Biotechnologies), following manufacturer's instructions. The prepared library comprised approximately 11,600 clones in *Escherichia coli* EPI300-T1 strain. The colonies were pooled into groups of five clones and arrayed in 25 96-well microtiter plates. Colonies of the metagenomic library were manually picked with sterile pipette tips, and cultivated in 96-well plates of 2 mL per well, filling each well with 0.2 mL of LB medium (1% tryptone, 0.5% yeast extract, and 1% NaCl) supplemented with 12.5 mg/mL chloramphenicol. Cells from five different colonies were pooled per well. These cultures were grown at 37°C for 24 h and used for functional screening, for storage at −80°C and to inoculate new cultures for DNA preparation for sequencing.

### Metagenomic library sequencing

**Figure 1:**
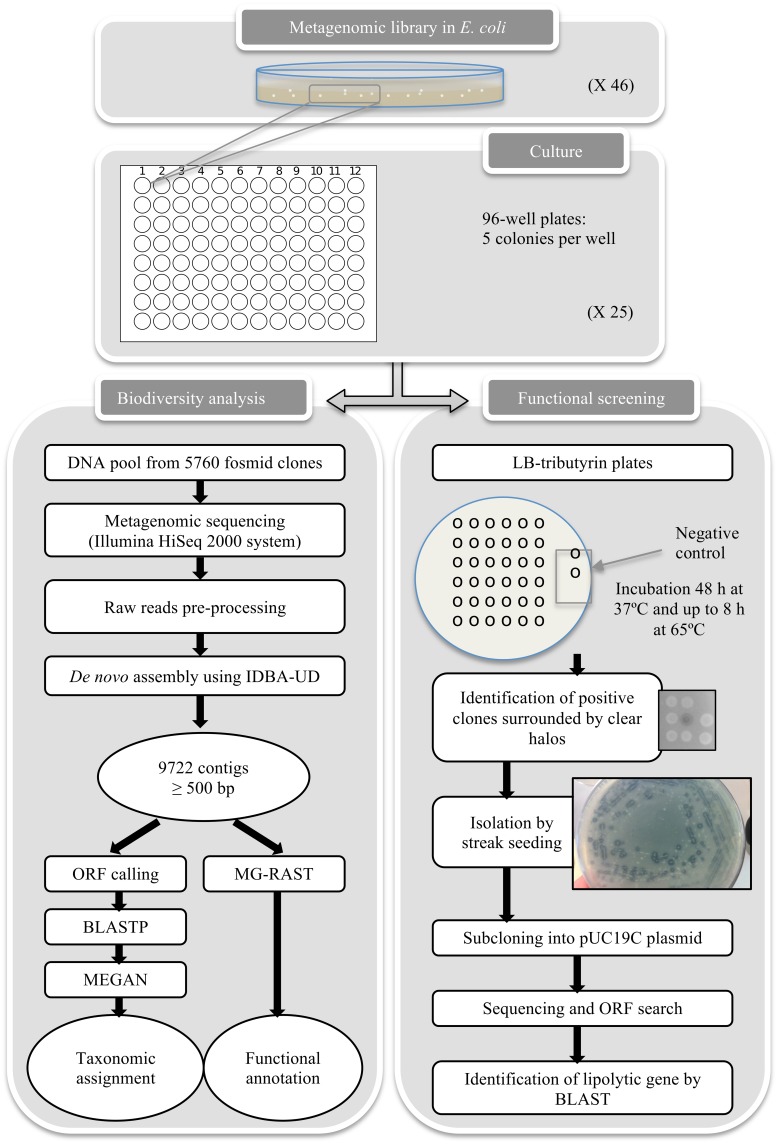
**A flowchart showing the steps of the functional screening procedure and bioinformatic analysis**.

Fosmid clones were grown overnight in liquid LB medium supplemented with 12.5 μg/mL chloramphenicol and 1X CopyControl Induction Solution (Epicentre) in order to induce to multicopy state. Twelve of the 25 96-well plates of the library were used one culture for each plate was run. 5 mL of medium were inoculated with 2 μl of each well and grown overnight. Fosmid DNA of each culture was extracted using FosmidMAX™ DNA Purification Kit (Epicentre) and pooled mixing the same DNA amount from each extraction. Subsequently, 5 μg of the pooled fosmid DNA was sequenced using Illumina HiSeq 2000 System at the Bioarray, S.L. (Alicante, Spain). A total of 11,982,436 reads with a read size of 100 bp were generated. A flow diagram of sequence processing is shown in Figure 1.

### Sequence pre-processing and assembly

The large-scale computational analyses were performed on a high performance computing cluster, The Supercomputing Centre of Galicia (CESGA). Reads with ambiguous bases (“Ns”), sequence duplicates and low-complexity sequences with DUST score < 7 were removed using PRINSEQ (Schmieder and Edwards, 2011). Reads matching to the cloning vector pCC1FOS (Genbank accession EU140751) and *E. coli* genome (NC_000913) were removed using Deconseq standalone (version 0.4.3) using 90% coverage and 94% identity filtering options (Schmieder and Edwards, 2011). Remaining reads were then assembled using IDBA-UD version 1.0.9 (Peng et al., 2012). Contigs shorter than 500 bp were discarded, leaving 9722 sequences for analysis. The raw sequencing reads and the assembled metagenome dataset have been deposited at the NCBI Short Read Archive under BioProject ID PRJNA294671 and accession number SRP063292.

### Sequence annotation and lipolytic genes screening

The putative Open Reading Frames (ORFs) were predicted from contigs ≥500 bp using MetaGeneMark Heuristic Approach (Zhu et al., 2010). Predicted ORFs were annotated by BLASTP (version 2.2.28+) against the NCBI protein (nr) database (updated on 1 June 2014) using an e-value 1e-05 and one best match was retained (−max_target_seqs 1). BLAST output files (in xml format) were imported into MEGAN (MEtaGenome ANalyzer) software (version 5.4.0) to perform taxonomic analysis using a Min score of 100, Top percent value 10%, Min support percent of 0 and Min support of 1 (Huson et al., 2007). For functional annotation, assembled contigs (≥500 bp) were uploaded onto publically available server MG-RAST (the Metagenomics Rapid Annotation using Subsystem Technology, version 3.5; Meyer et al., 2008). Protein features predicted by MG-RAST were assigned to functional categories using Subsystems database (e-value 1e-05, % identity cut-off of 60% and minimum alignment length 15). Annotated contigs can be accessed through MG-RAST website under the Project ID 4570559.3.

Alpha diversity calculation was performed on assembled data using the MG-RAST server. Datasets publicly available in MG-RAST of hot springs from Colombia (4449206.3), China (4530144.3), Russia (4544453.3), Italy (4529716.3), Iceland (4530143.3), and USA (4529719.3), were used for the comparison (Table 1).

**Table 1:** **Alpha diversity estimates for various hot springs around the world**.

**Sample location**	**T(°C)**	**pH**	**MG-RAST accession no**	α-Diversity^*^	**Reference**
China	65	7.0	4530144.3	457.729	Menzel et al., 2015
Russia	61–64	5.8–6.0	4544453.3	615.968	
Italy	76	3.0	4529716.3	86.121	
Iceland	85–90	5.0	4530143.3	196.142	
USA	92	3.0–4.0	4529719.3	117.640	
Colombia	29	2.7	4449206.3	467.609	Jiménez et al., 2012
Spain	76	8.2	4570559.3	330.865	This study

* *Alpha diversity, number of distinct species in a sample, was estimated using the MG-RAST server (Meyer et al., 2008)*.

To identify lipase and esterase genes in fosmid metagenome, predicted ORFs from contigs (≥500 bp) were compared to 246 lipase protein sequences using BLASTP and e-value 1e-05. The lipase and esterase protein sequences were retrieved from UniProtKB/Swiss-Prot (reviewed) database (http://www.uniprot.org/) against query “EC:3.1.1.1” and “EC:3.1.1.3” and downloaded in a fasta format in March 2015. Sequences associated with lipolytic genes according to UniProt database BLAST results were extracted for further BLAST against NCBI nr database.

### Functional screening

The metagenomic library was screened for fosmid clones showing lipolytic activity in plates containing 1% tributyrin as substrate (Glogauer et al., 2011). Drops of 5 μL of each well of the 25 96-well plates of the library were used to inoculate tributyrin plates. The plates were incubated for 2 days at 37°C and then further incubated at 65°C up to 8 h. The appearance of clear halos around the drops indicated the hydrolysis of tributyrin. The clone that showed a clear halo the earliest was selected for subcloning and expression, and was isolated by streak seeding on tributyrin plates in order to obtain single colonies surrounded by clear halos (Figure 1).

### Subcloning and sequence analysis

Fosmid from selected positive clone FOS4 was isolated using the FosmidMAX DNA purification kit (Epicentre Biotechnologies) and digested with *Sph*I and *Eco*RI. Fragments were subcloned into pUC19 plasmid by using *Sph*I and/or *Eco*RI restriction sites and plated on LB-tributyrin plates supplemented with 100 μg/mL ampicillin.

The insert present in positive colonies (those surrounded by clear halos) was sequenced by the primer-walking technique using the sequencing service of Sistemas Genómicos (Valencia, Spain). The ORFs in the DNA sequence were detected by the online tool MetaGeneMark (Besemer and Borodovsky, 1999; Zhu et al., 2010) prediction software and similarity searches were carried out with BLASTP (Altschul et al., 1990). The ORF3 showed similarity to lipolytic enzymes and was selected for further experiments.

The amino acid sequence of the new identified lipolytic protein was aligned using ClustalW (Larkin et al., 2007) with five top hits from BLASTP search together with 28 lipolytic enzymes sequences from previously established groups (I–VIII; Arpigny and Jaeger, 1999). The resulting alignment was edited in Jalview (Waterhouse et al., 2009; Zhu et al., 2010) and the phylogenetic tree was constructed in MEGA6 (Tamura et al., 2007) using a p-distance model, complete deletion of gaps and the neighbor-joining method with 1000 bootstrap replications. The protein encoded by ORF3 turned to be an esterase and the sequence of the gene has been submitted to the GenBank database under accession number KR045603.

Multiple sequence alignments of protein encoded by ORF3 with selected family VIII esterases were performed by ClustalW. Signal peptide search was performed by Signal P 4.0 program. Physicochemical parameters of the protein were predicted by ProtParam online tool (Gasteiger et al., 2005).

The gene encoding the new lipolytic enzyme was named *LOB4Est* and cloned in the YEpFLAG-1 plasmid (Eastman Kodak Company) for heterologous expression in *S. cerevisiae* BJ3505 (pep4::HIS3 prb- 1.6R HIS3 lys2-208 trp1-101 ura3-52 gal2 can1; Eastman Kodak Company). The primers YFF4A3F (AAAAGAGACTACAAGGATGACGATGACAA Gagccgcccgcgtaccg) and YFF4A3R (TGGGACGCTCGACGGATCAGCGGCC GCTTAggcgcagccgagttcctcgc) were used to amplify the sequence of *LOB4Est*. The upper case letters correspond to regions of homology to the cloning vector YEpFLAG-1 that allowed the cloning of the gene into the expression vector by homologous recombination. PCR cycling conditions were: initial denaturation (95°C, 5 min); followed by 30 cycles of denaturation (95°C, 30 s), annealing (64°C, 30 s), extension (72°C, 1 min 10 s); and a final cycle of 72°C for 7 min; Pfu DNA polymerase (Thermo Scientific) and PCR buffer containing 4% DMSO were used. Cells of *S. cerevisiae* BJ3505 were co-transformed by the lithium acetate procedure (Ito et al., 1983) with the PCR product and the YEpFLAG-1 plasmid, previously linearized by digestion with *Eco*RI and *Sal*I (Takara), and plated in a tryptophan-free complete medium for selection of transformants containing the recombinant plasmid YEpFLAG1-LOB4Est. Firstly, the clones were confirmed by determination of esterase activity in extracellular medium as described below. Then, the recombinant plasmid was extracted from the yeast cells using the Spin CleanTM Plasmid Miniprep Kit (Metabion) and propagated into the *E. coli* strain Ecos Blue (Novagen) to obtain enough recombinant DNA to verify the correctness of the construction by sequencing.

### Culture conditions

For expression and characterization of the recombinant protein, the recombinant strain Sc-LOB4Est was grown in YPHSM medium (8% bactopeptone, 1% yeast extract, 3% glycerol, and 1% dextrose, w/v), designed by Eastman Kodak Company to improve stability of secreted recombinant proteins.

For biochemical characterization, the recombinant strain Sc-LOB4Est was grown for 3 days at 30°C in 50 mL screw-capped glass tubes filled with 2 mL of YPHSM medium, at 200 rpm.

For analysis of expression the recombinant strain was grown in Erlenmeyer flasks, filled up to 20% volume with YPHSM. Cultures were initiated by the addition of 1:20 volume of 48 h pre-culture in CM-trp, and grown at 30°C and 200 rpm.

### Cell fractionation

Subcellular location of the recombinant enzyme, to verify its correct secretion, was determined by measuring lipolytic activity in different cell compartments: extracellular, periplasmic, cytoplasmic, and membrane bound. The fractions were prepared by the method described in Becerra et al. (2001).

### Esterase activity

Lipolytic activity was determined by a spectrophotometric method using *p*-nitrophenyl laurate as substrate (Fuciños et al., 2005). Briefly, 400 μL of activity buffer (150 mM pH 7.5 at 40°C Tris/HCl, 40 mM CaCl_2_) and 50 μL of *p*-nitrophenil laurate stock solution (25 mM) were incubated for 10 min at 40°C. The addition of 50 μL of extracellular medium containing the recombinant enzyme initiated the reaction, which was stopped after 20 min of incubation with 125 μL of cold 1 M Na_2_CO_3_. The tubes were placed immediately on ice for 15 min and then the precipitate was eliminated by centrifugation at 14,000 rpm for 10 min. A_400_ of the supernatant was measured. A blank was prepared using water instead of enzyme solution. One activity unit was defined as the amount of enzyme that produced 1 μmol of *p*-nitrophenol/min under standard assay conditions. The activities were expressed in U/L of culture medium or in U/mg of protein.

Protein concentration was measured using the Bradford Assay (Bio-Rad) and bovine serum albumin as standard.

### Biochemical characterization of the recombinant enzyme

Small cultures of 2 mL were performed. Supernatant was separated from cells by centrifugation, 13,000 rpm for 2 min, and used for biochemical characterization.

Substrate preference was determined using as substrates *p*-nitrophenyl esters with different side chain lenght: *p*-nitrophenyl butyrate (pNP4), *p*-nitrophenyl hexanoate (pNP6), *p*-nitrophenyl octanoate (pNP8), and *p*-nitrophenyl laurate (pNP12). The kinetic parameters (Km and Vmax) were determined measuring the initial conversion rates using pNP12 as substrate at different concentrations (0.002–2.5 mM).

The dependence of lipolytic activity on temperature was studied at different temperatures ranging from 40 to 80°C. Buffer pH (7.5) was adjusted at each temperature. The dependence of lipolytic activity on pH was studied at 40°C using different buffer systems at values of pH ranging from 4.5 to 8.5: buffer sodium acetate/acetic acid (pH 4.5, 5.0, and 5.5), buffer Tris/maleic acid (pH 6.0, 6.5, 7.0, and 7.5), and buffer Tris/HCl (pH 7.5, 8.0, and 8.5). In both cases, the reaction time was reduced to 10 min, to minimize the potential effects of extreme temperature or pH in protein stability.

Thermostability was studied by measuring the residual activity after incubation of samples of extracellular medium containing recombinant enzyme at 50 and 60°C. Samples were taken at prefixed time points.

### β-lactamase activity

β-lactamase activity was determined by a spectrophotometric method using nitrocefin [3-(2, 4 dinitrostyrl)-(6R,7R-7-(2-thienylacetamido)-ceph-3-em-4-carboxylic acid, E-isomer] (Calbiochem) as substrate (Jeon et al., 2011). Ten microliter of extracellular medium containing recombinant enzyme were incubated with 150 μL of reaction buffer (0.1 M sodium phosphate, 1 mM EDTA, pH 7.0) at 40°C for 5 min. The reaction was initiated by the addition of 40 μL of a stock solution of 5 mM nitrocefin, to reach a final concentration of 1 mM, and the rate change of A_486_ was measured. A blank was prepared using water instead of enzyme solution, and a control was prepared using extracellular medium from a culture of the *S. cerevisiae* BJ3505 strain transformed with empty vector YEpFLAG-1. The activity was expressed in relative values.

### Polyacrylamide gel electrophoresis and western blotting

SDS-PAGE and Western blotting were performed by the procedure described in Becerra et al. (1997) but using anti-FLAG-M2 monoclonal antibody (Sigma) and goat anti-mouse IgG HRP conjugated (Santa Cruz Biotecnology).

## Results and discussion

The Lobios hot spring is one of the highest temperature (>76°C) and alkaline pH (>8.2) of the Galician region. This hot spring is situated near the Lobios town and on the riverbank of Río Caldo River, 70 km from Ourense (Spain) and 6 km from Portugal (GPS 41.86113, −8.1062). The thermal spring is located underground of the Spa Hotel and Resort (Figure S1 in Supplementary Material) and tapped by a borehole, which provides water to the Spa. The water is bicarbonate-chloride-fluoride low mineral type (Ramírez Ortega et al., 2007). Little is known regarding the microbial communities and biotechnological potential of this thermoalkaline spring. The aim of this work is to construct a metagenomic fosmid library of this hot spring, as well as evaluate, by sequence-based and functional approaches, its biodiversity and usefulness for the screening and characterization of novel thermostable and alkaline-tolerant lipolytic enzymes (Figure 1).

### Metagenomic library construction

A fosmid library consisting of approximately 11,600 clones was constructed from metagenomic DNA isolated from the Lobios hot spring water, as described in Materials and Methods.

### Fosmid library sequencing and assembly

A total of 5,416,438 reads were generated after removing of the low-quality reads and sequences corresponding to *E. coli* genome and fosmid vector. Over 82% of these reads were assembled into 9722 contigs >500 bp, with the N50 length of 2.9 kb and the total contig length of 17.9 Mb. A total of 23,207 ORFs were predicted from the assembly. A summary of Illumina sequencing and contig assembly results is presented in Table S1.

### Taxonomic diversity of the fosmid library

The MG-RAST alpha diversity analysis showed that the diversity within the library was higher (330.865 distinct species) when compared with diversity of high temperature (76–92°C) hot spring metagenomes from Italy (86.121 species), USA (117.640 species), and Iceland (196.142 species), and slightly lower than a low temperature (29°C) hot spring metagenome from Colombia (467.609 species) and moderate temperature (61–65°C) hot spring metagenomes from China (457.729 species) and Russia (615.968 species; Table 1). An inversely proportional relationship between hotspring temperature and degree of microbial diversity has been previously reported (Cole et al., 2013).

**Figure 2:**
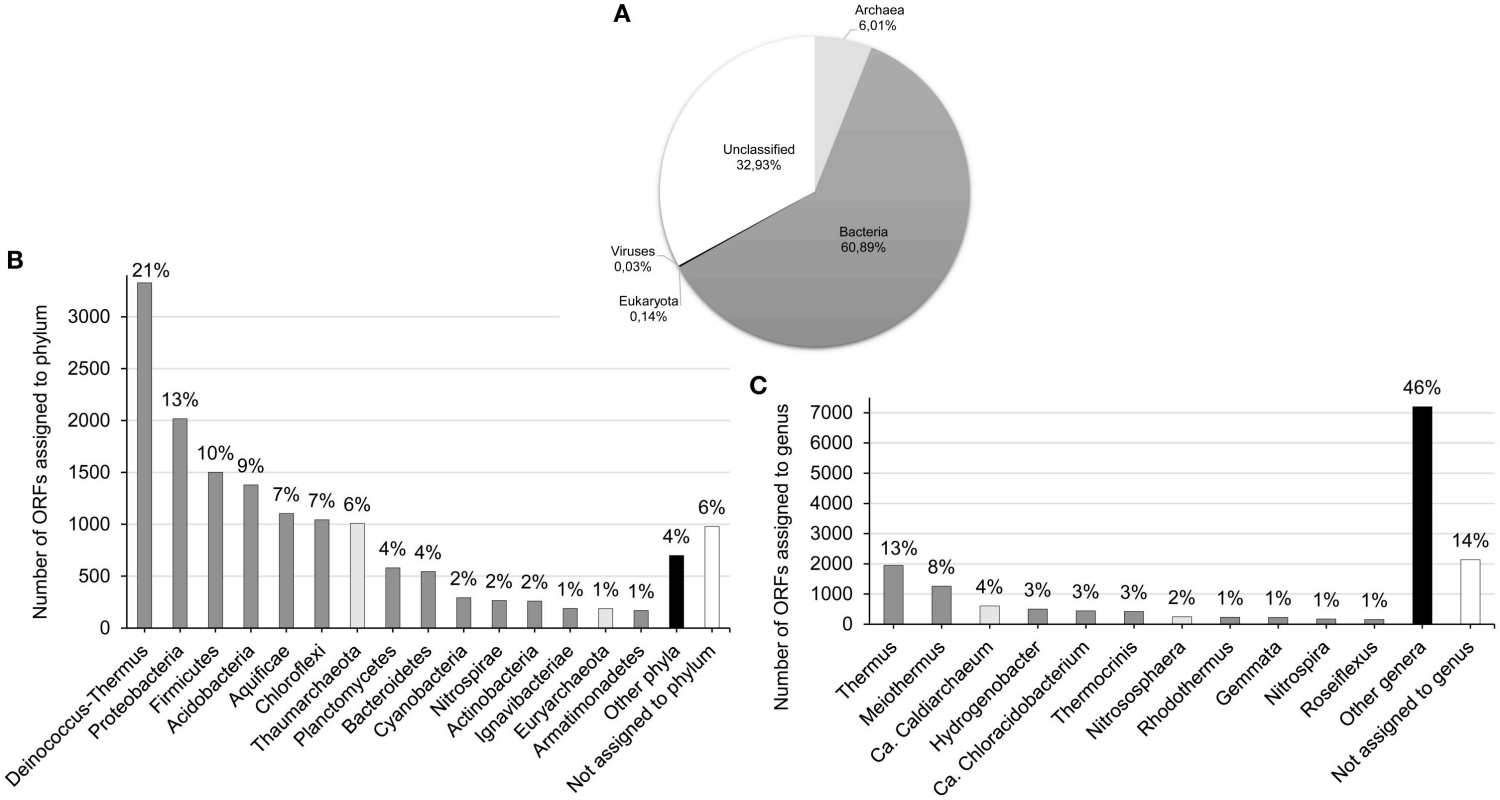
**Taxonomic classification of ORFs predicted from contigs. (A)** All ORFs (*n* = 23.207) predicted from contigs (≥500 bp) at the superkingdom level. Predicted ORFs were compared using BLAST to the NCBI protein database and assigned to taxa using MEGAN with a minimum bit-score of 100 and e-value 1e-05. Over 67% (15,565) ORFs were assigned to Bacteria, Eukaryota, Achaea, and Viruses, while < 33% remained unclassified. **(B)** ORFs assigned at the phylum level. Of 15,557 ORFs assigned by MEGAN to Bacteria, Eukaryota, and Achaea, 14,577 (94%) could be classified at the phylum level. Bacterial phyla are colored in dark gray and achaeal are represented by light gray bars. Phyla with abundance < 1% were collapsed into the “Other phyla” category (black bar). **(C)** ORFs assigned at the genus level. Of 15,557 ORFs assigned by MEGAN to Bacteria, Eukaryota, and Achaea, 13,419 (86%) could be classified at the genus level. Bacterial genera are colored in dark gray and archaeal are represented by light gray bars. Genera with abundance less < 1% were collapsed into the “Other genera” category (black bar).

The results of predicted ORFs analysis showed that the majority of sequences from the Lobios hot spring metagenome were assigned to Bacteria (61%) and Archaea (6%), and a small portion of sequences was classified as Eukaryota and Viruses (Figure 2A). Less than 33% of the sequences could not be classified and may represent previously uncharacterized microorganisms. Full results can be found in Table S2.

The majority of the classified protein sequences could be affiliated with 29 prokaryotic and 5 archaeal phyla. The dominant prokaryotic phyla among the classified sequences were Deinococcus-Thermus (21%), Proteobacteria (13%), Firmicutes (10%), Acidobacteria (9%), Aquificae (7%), and Chloroflexi (7%) (Figure 2B). Members of these phyla are commonly observed in thermal springs around the world (Hall et al., 2008; Pagaling et al., 2012; Bowen de León et al., 2013; Wang et al., 2013). Archaea superkingdom was overrepresented by Thaumarchaeota phylum, which accounted for 6% of all classified sequences and 72% of all archaeal sequences. The high occurrence of Thaumarchaeota in the archaeal fraction has been previously reported in slightly alkaline springs from Kamchatka and China (Huang et al., 2011; Wemheuer et al., 2013). The second abundant archaeal phylum was methane-producing Euryarchaeota, accounting for 1% of all classified sequences. The predominance of Bacteria over Archaea in the Lobios hot spring may be influenced by its alkaline pH. Menzel et al. noticed that relative abundance of Archaea in hot springs is higher in low pH and high temperature environments (Menzel et al., 2015).

The most abundant genera among the classified sequences were: Thermus (13%), Meiothermus (8%), Candidatus Caldiarchaeum (4%), Hydrogenobacter (3%), Candidatus Chloracidobacterium (3%), Thermocrinis (3%), Nitrososphaera (2%), Rhodothermus (1%), Gemmata (1%), Nitrospira (1%), and Roseiflexus (1%) (Figure 2C). Analysis of the most abundant species (Table S3) revealed that the high-temperature alkaline Lobios spring was dominated by thermophilic, neutrophilic, or alkaliphilic organisms, a mix of aerobes and anaerobes, chemoorganotrophs and chemolithotrophs, heterotrophs and autotrophs. The most abundant heterotrophs and chemoorganotrophs belonged to Thermus (*Thermus scotoductus*, Thermus sp. NMX2.A1, *Thermus igniterrae, Thermus islandicus*, and *Thermus thermophilus*) and Meiothermus (*Meiothermus timidus, Meiothermus Silvanus*, and *Meiothermus ruber*; Table S3). Thermus and Meiothermus are commonly found in hot springs worldwide, occurring throughout a wide range of temperatures and pHs (Purcell et al., 2007; Boomer et al., 2009; Costa et al., 2009; Vick et al., 2010; Mardanov et al., 2011; Tobler and Benning, 2011; Bowen de León et al., 2013; Wang et al., 2013). Abundance of heterotrophic bacteria may be probably due to a high content of dissolved organic carbon in Lobios hot spring water. Although one previous study reveals the presence of soluble organic compounds in several hot springs in Galicia (González-Barreiro et al., 2009), the Lobios hot spring is not among them, and we have not found publications about its organic matter content.

Among chemolithotrophs found in the metagenome there were nitrifying, sulfur-oxidizing, hydrogenotrophic and methanogenic species. During the chemolithotrophic growth, microorganisms obtain energy by using carbon dioxide as an electron acceptor to oxidize hydrogen to other compounds (hydrogenotrophic) such us methane (methanogenic), and hydrogen sulfide, or elemental sulfur to sulfuric acid (sulfur-oxidizing). Hydrogen- and sulfur-oxidizing species were the most abundant among chemolithotrophs. The hydrogen-oxidizing species in the Lobios hot spring were derived from *Caldiarchaeum subterraneum* (potential hydrogenotroph; 3.9%), *Hydrogenobacter thermophilus* (3.2%), *Thermomicrobium roseum* (0.8%), *Aquifex aeolicus* (0.4%), and *Ammonifex degensii* (0.3%). The sulfur-oxidizing bacteria and archaea belonged mostly to *Thermus* (*Thermus scotoductus, Thermus islandicus*; 6.7%), *Chlorobiaceae* (0.4%), *Sulfobacillus* (0.1%), *Thioalkalivibrio* (0.1%), and Crenarchaeota (mainly *Sulfolobus* and *Acidianus*; 0.1%). Among the hydrogen- and sulfur-oxidizing bacteria were *Thermocrinis* (*Thermocrinis ruber, Thermocrinis albus*; 2.7%), *Methylococcus capsulatus* (0.5%), *Hydrogenivirga* sp. (0.4%), and *Dethiobacter alkaliphilus* (0.1%) (Brock et al., 1972; Sorokin et al., 2008).

Nitrifying chemolithotrophic microorganisms generate energy by the oxidation of ammonia to nitrite (ammonia oxidizers) or nitrite to nitrate (nitrite oxidizers). Nitrification is a key part of global nitrogen cycling performed by bacteria and archaea (Bai et al., 2012). Nitrifying organisms in the Lobios metagenome belonged to the phylum Proteobacteria, Thaumarchaeota, Chloroflexi, Nitrospirae, and Nitrospinae. Among them were nitrite-oxidizing members of bacterial genera Nitrospira (*Nitrospira defluvii*; 1.1%), *Nitrolancea* (0.3%), *Nitrosococcus* (0.2%), *Nitrosomonas* (0.1%), *Nitrococcus* (0.1%), *Nitrospina* (0.1%) (Lücker et al., 2010) and ammonia-oxidizing thermophilic archaeons *Nitrososphaera gargensis* (1.6%) and *Nitrosopumilus* (0.2%) (Walker et al., 2010; Park et al., 2012).

Methanotrophic bacteria play an important role in global carbon cycles by oxidation of one-carbon compounds, mostly methane (Hanson and Hanson, 1996). The most abundant methanotrophs in the metagenome belonged to Proteobacteria, mostly Methylococcaceae (*Methylococcus capsulatus*; 0.6%) and Methylothermaceae (*Methylohalobius crimeensis*; 0.3%) family. Other abundant methanotroph present in the metagenome was “*Candidatus* Methylomirabilis oxyfera” (0.6%), a member of the candidate phylum NC10.

Although the Lobios thermal water was not exposed to light at the point of sampling, several thermophilic photosynthetic microorganisms were detected in the Lobios metagenome. Their origin is uncertain. These included the phototrophic bacteria from phylum Acidobacteria (*Chloracidobacterium thermophilum*; 2.9%), Cyanobacteria (1.9%), Chloroflexi (*Roseiflexus*; 1%), Chlorobi (0.4%), and some Firmicutes (heliobacteria; 0.07%). *C*. *thermophilum* was one of the most abundant species in the metagenome. It is a moderately thermophilic chlorophotoheterotroph that produces chlorosomes, which enable the cell to generate chemical energy from light (Garcia Costas et al., 2012; Tank and Bryant, 2015). However, it is not known if these phototrophic bacteria were photosynthetically active in the 76°C Lobios spring. To date, the highest temperature at which photosynthesis has been reported to take place is 75°C (Meeks and Castenholz, 1971).

### Functional diversity

**Figure 3:**
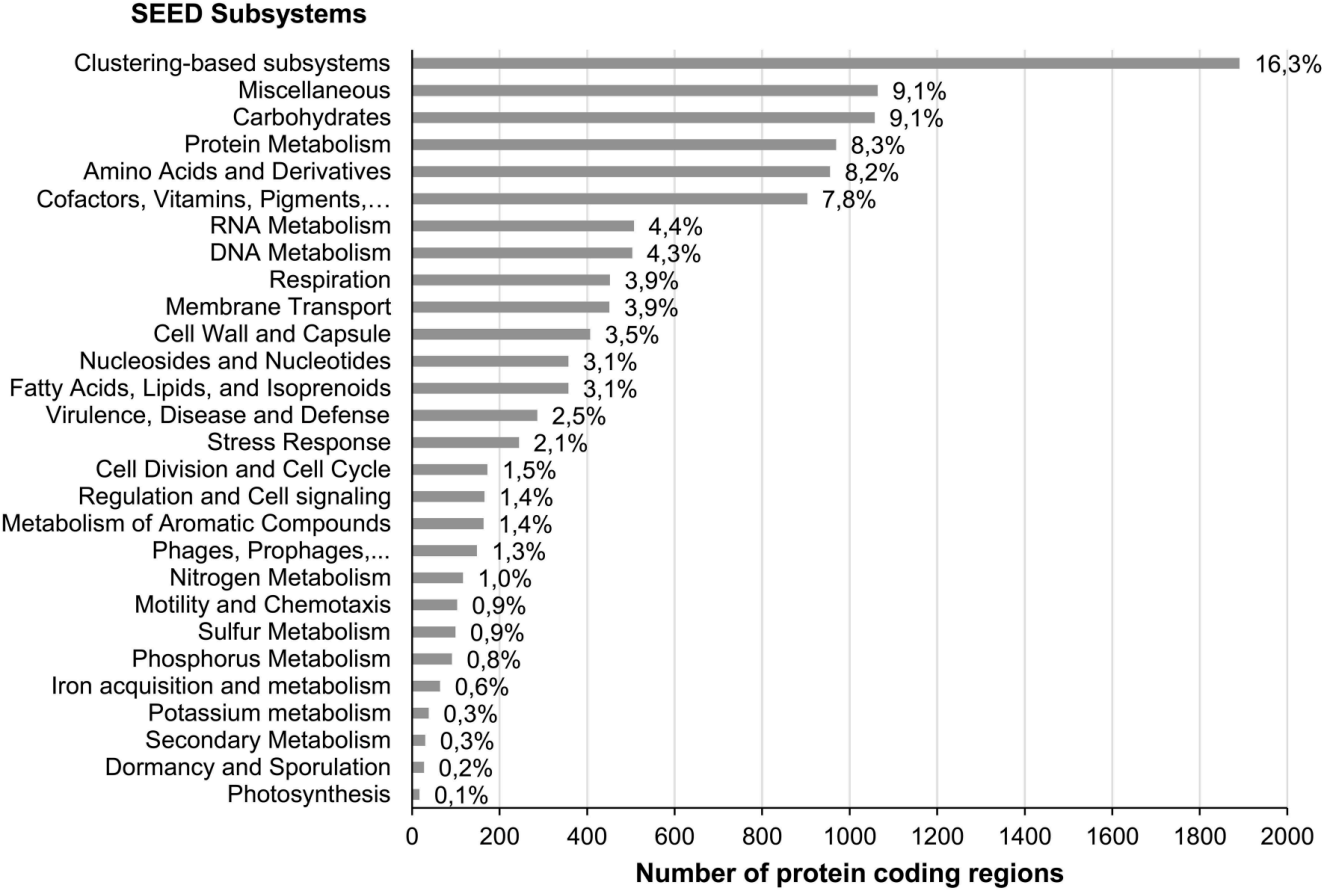
**Functional classification of contigs using MG-RAST**. Of 17,994 protein coding regions predicted from contigs by MG-RAST, 65% (11,636) were assigned by MG-RAST to SEED functional categories (Subsystems).

The functional annotation was carried out based on SEED subsystems classification. Subsystems are sets of proteins that share similar functions (Overbeek et al., 2005). Of the 17,994 protein coding regions predicted by MG-RAST calling system from the assembled contigs, 11,636 (65%) were assigned to SEED functional categories (Figure 3).

Among the 28 SEED subsystems present at level 1 (Figure 3, Table S4), the subsystem Clustering-based subsystems (1891, 16.3%) was the largest category, followed by Miscellaneous (1064, 9.1%), Carbohydrates (1057, 9.1%), Protein Metabolism (969, 8.3%), Amino Acids and Derivatives (955, 8.2%), and Cofactors, Vitamins, Prosthetic Groups, Pigments (903, 7.8%). A clustering-based subsystem is a subsystem in which there is functional coupling evidence among the proteins, but their exact roles in the metabolic pathways are yet unknown. The prevalence of this category in the metagenome reveals the lack of knowledge that still exists about the function of a large part of the microbial proteome, and reinforces the idea that there is a good chance to find new activities by functional screening of this library.

Analysis of the subsystems at level 3 (Table S4) revealed high abundance of genes involved in different carbon assimilation pathways. Sequences associated with one-carbon metabolism: a folate-binding protein YgfZ, that plays a folate-dependent regulatory role in one-carbon metabolism (Teplyakov et al., 2004) and genes associated with serine-glyoxylate cycle were the most abundant and accounted together for 4.1% (482 ORFs) of all annotated sequences. The serine-glyoxylate cycle is the pathway for single-carbon (C1) compounds assimilation, such as methane and methanol, and it is found in many methylotrophic bacteria (Chistoserdova, 2011). However, the key enzymes for methylotrophic metabolism, that is the methane monooxygenase and methanol dehydrogenase encoding genes, were not found.

The presence of genes involved in methanogenesis was detected in the metagenome. Methanogens produce methane by reducing CO_2_, acetate or methyl group-containing compounds (Ferry, 2011). The key enzymes involved in methanogenesis included carbon monoxide dehydrogenase, formate dehydrogenase, heterodisulfide reductase and formyl-methanofuran dehydrogenase.

The key enzymes involved in autotrophic CO_2_ fixation pathways were associated with photorespiration, reductive tricarboxylic acid cycle and Calvin-Benson cycle (Table S4). The key genes of CO_2_ fixation pathways present in thermophilic Archaea and some bacteria (Berg et al., 2007, 2010; Huber et al., 2008), the 3-hydroxypropionate/4-hydroxybutyrate cycle (propionyl-CoA carboxylase) and bicarboxylate/4-hydroxybutyrate cycle (pyruvate synthase and phosphoenolpyruvate carboxylase) were also detected in the metagenome. Sequences associated with photosynthesis coded for enzymes that participate in the proteorhodopsin and bacteriorhodopsin biosynthesis pathways. The metagenome contained two ORFs with homology to RuBisCo genes.

The presence of TCA cycle and terminal oxidase complexes (NADH-ubiquinone oxidoreductase, succinate dehydrogenase, cytochrome *c* reductase, and cytochrome *c* oxidase) in the metagenome suggests presence of organisms that can respire oxygen. The metagenome also contained genes associated with anaerobic metabolism (anaerobic reductases and hydrogenases). Among anaerobic reductases and hydrogenases there were genes that allow anaerobic respiration using as terminal electron acceptor nitrate (respiratory nitrate reductase), nitrite (nitrite reductase), nitric oxide (nitric-oxide reductase), DMSO (dimethyl sulfoxide reductase), heterodisulfide (heterodisulfide reductase), arsenate (arsenate reductase), fumarate (fumarate reductase), tetrathionate (tetrathionate reductase), sulfite (dissimilatory sulfite reductase), and formate (formate hydrogenlyase).

Analysis of the subsystems at the “function” level (Table S4) revealed the presence of proteins with potential biotechnological interest. For example, we found genes encoding for xylanases, galactosidases, proteases, and lipases. Thermostable variants of these kinds of enzymes are currently at high demand in industry.

Remarkably, over 30% of genes in the Lobios metagenome could not be identified using bioinformatics methods, and therefore can only be found by functional screening of the library.

### Sequence-based and functional lipolytic activity screening

Lipase genes were retrieved from the metagenomic fosmid library using the sequence-based approach described in Section Sequence Annotation and Lipolytic Genes Screening. As shown in Table S5, 11 ORFs showed homology to lipolytic enzymes. They belong to the genera Meiothermus (4), Thermus (2), Clostridium (1), Hydrogenivirga (1), Bdellovibrio (1), and uncultured organisms (2).

In parallel, the clones of the metagenomic library were screened for lipolytic activity in LB-tributyrin plates and six positive clones with clear halos indicating lipolytic activity were found after incubation during 48 h at 37°C followed by up to 8 h at 65°C. The frequency of lipolytic positive clones obtained, 0.05%, was in the order of magnitude of other previously reported metagenomics study (Martínez-Martínez et al., 2013). Moreover, taking into account that about half of thermophilic sequences are not actively expressed in mesophilic hosts (Leis et al., 2015), the frequency of positive clones obtained in the function-based screening was as expected in function of the frequency obtained in the sequence-based metagenome mining.

**Figure 4:**
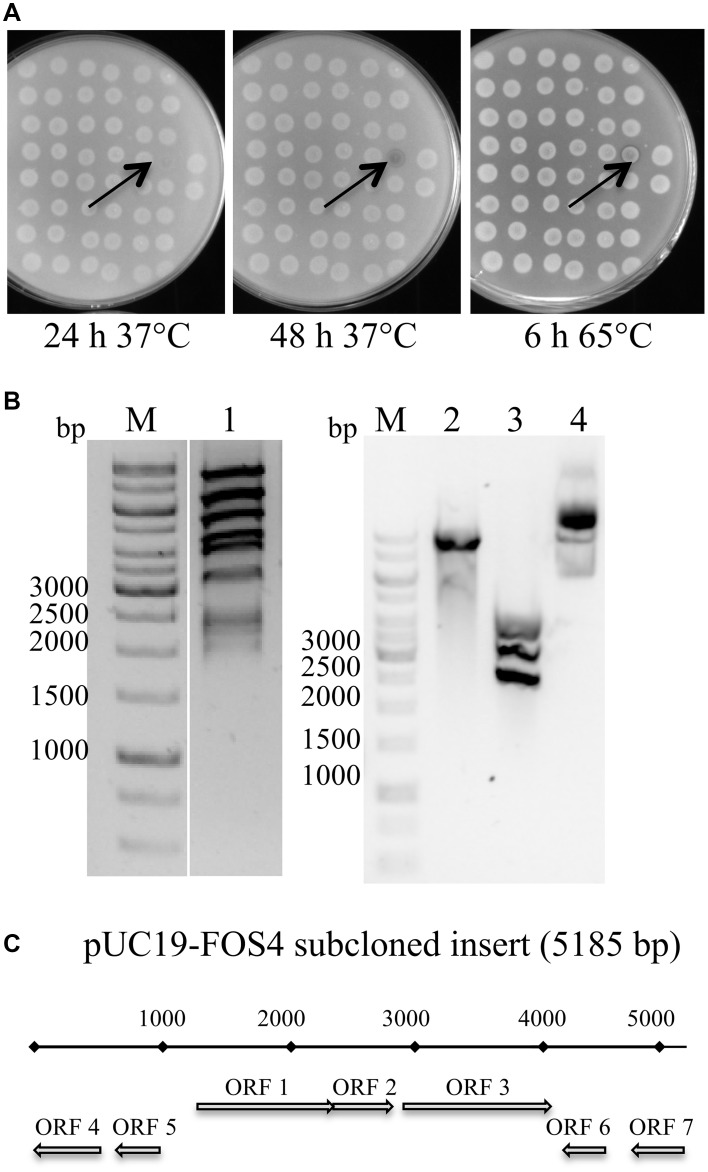
**Selection, subcloning, and identification of the *LOB4Est* gene: (A)** Images of the screening plate containing the lipolytic positive clone FOS4 after incubation for 24 and 48 h at 37°C, and 6 h at 65°C. **(B)** Analysis by electrophoresis in agarose gels (0.8%) of: 1- digestion by SphI and EcoRI of fosmid FOS4 (2686 bp); 2- digestion by EcoRI of subclone in plasmid pUC19 (2628 bp); 3- digestion by SphI of subclone in plasmid pUC19; 4- subclone in plasmid pUC19 undigested. M: GeneRuler 1kb DNA Ladder (Fermentas). GelGreen stain. **(C)** Physical map of the insert of FOS4A subcloned into pUC19. Best BLASTP hits are shown in Table 2. The arrows into **(A)** point to the FOS4 clone. The arrows into **(C)** indicate the positions of the ORFs.

The clone that showed a clear halo in the shortest incubation time, FOS4, was selected for further experiments (Figure 4A).

### Identification of the lipolytic ORF

**Figure 5:**
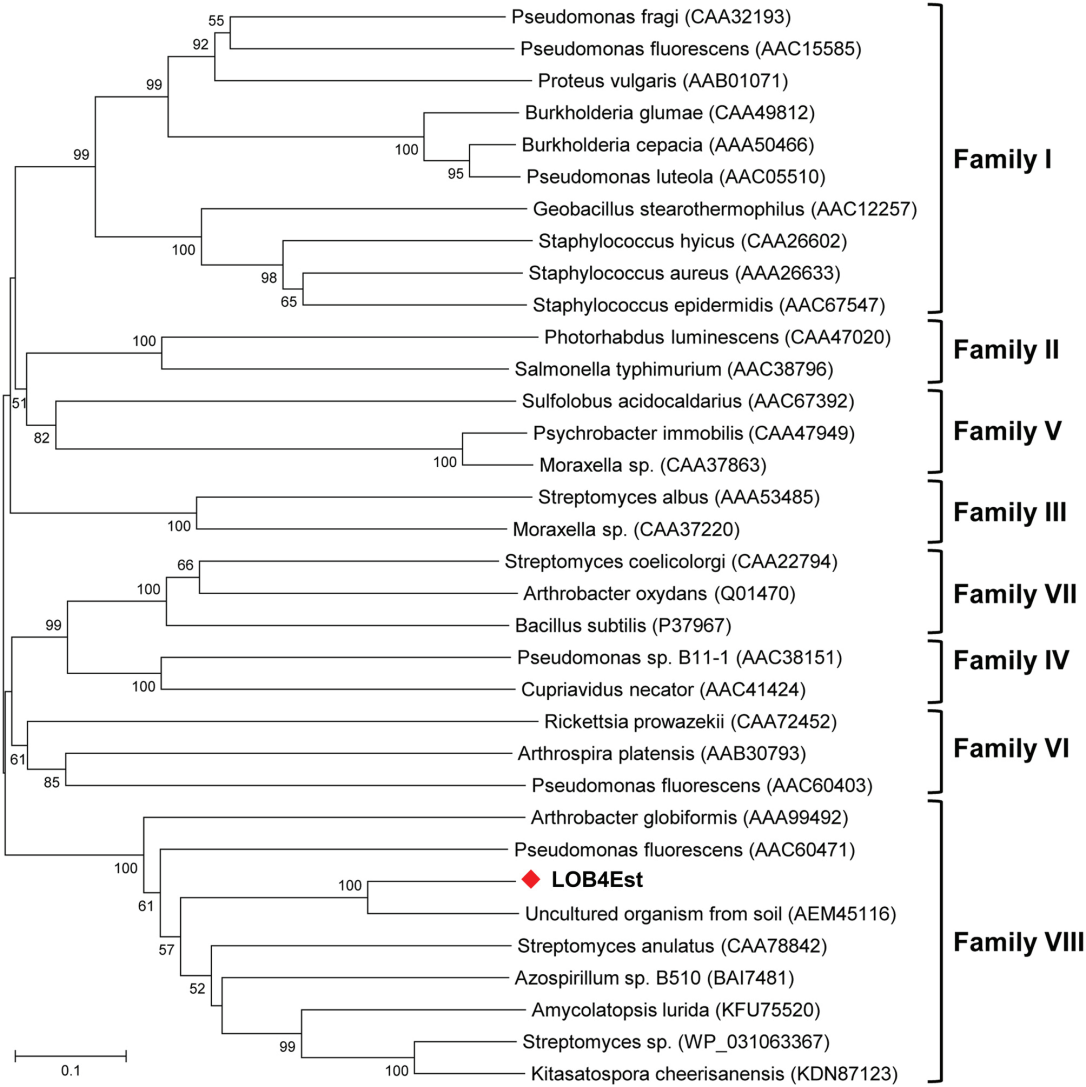
**Neighbor-joining phylogenetic tree based on amino acid sequence of the LOB4Est (shown in bold) and known members of lipase families I-VIII (Arpigny and Jaeger, 1999)**. GenBank accession numbers of the reference sequences are shown in parentheses. The tree was drawn using MEGA software, with p-distance model and 1000 bootstrap replications. Only bootstrap values >50 are shown.

The fosmid FOS4 was restriction digested, yielding DNA fragments of 2–10 kb (Figure 4B) that were subcloned into pUC19. The subclone showing lipolytic activity was sequenced and the insert size was about 5.2 kb. Seven ORFs were predicted from the insert (Figure 4C, Table 2). Although annotated ORFs were affiliated with different bacterial species, three of them matched with Clostridia, suggesting that the cloned insert could originate from a yet undescribed member of Clostridia. The ORF3 showed the highest amino acid similarity (99% query coverage and 64% identity) to the esterase Est8, isolated by functional metagenomics from soil in Germany (Nacke et al., 2011). The gene associated to ORF3, named *LOB4Est*, was therefore identified as the gene conferring lipolytic activity to the FOS4 clone. Phylogenetic analysis determined that the protein LOB4Est belongs to family VIII esterases (Figure 5), according to the classification of lipolytic enzymes by Arpigny and Jaeger (Arpigny and Jaeger, 1999). *LOB4Est* was not among the 11 lipase genes detected in the metagenomic library using the sequence-based approach (Table S5). However, there were 77 singletons that matched the *LOB4Est* sequence, indicating that more deep sequencing of the library would likely detect the enzyme.

**Table 2:** **Best hits by BLASTP search against non-redundant protein (nr) database for ORFs detected in the insert from FOS4A subcloned in pUC19**.

**ORF (bp)**	**Possible function and microorganism**	**Accession number**	**% Query coverage**	**% Identity**
ORF 1 (1254)	hypothetical protein, *Desulfotomaculum* sp. BIC-A1/1_c6	WP_034102674	84	44
ORF 2 (459)	alkyl hydroperoxide reductase, *Cyanothece* sp. PCC 7425	WP_012629091	80	48
ORF 3 (1149)	esterase Est8, uncultured organism from soil	AEM45116	99	64
ORF 4 (513)	O-phosphoserine sulfhydrylase, *Longispora albida*	WP_018348134	100	54
ORF 5 (339)	ModE family transcriptional regulator, *Acetohalobium arabaticum*	WP_013278309	98	43
ORF 6 (327)	hypothetical protein, *Porphyromonas* sp. COT-290_OH3588CRE	KGN97422	56	32
ORF 7 (402)	hypothetical protein, *Clostridiales* bacterium VE202-07	WP_024726181	100	55

Lipolytic enzymes of family VIII are poorly characterized hitherto. Their physiological function is still unknown, but they are considered valuable biocatalysts since they are active, and highly enantioselective, on industrially important or rare compounds that are not accepted as substrates by standard lipolytic enzymes. Some examples of these compounds are: bulky esters of tertiary alcohols for pharmaceutical applications (Petersen et al., 2001; Wagner et al., 2002; Rashamuse et al., 2009), (+)-methylacetate for the food industry and the production of perfumes (Elend et al., 2006), aryl-carboxylic acid esters for the production of aromas or preservatives, removal of environmental pollutants or the selective removal of protecting groups (Takehara et al., 2012). Besides, family VIII enzymes often show a remarkably stable and even enhanced activity in the presence of organic solvents (Elend et al., 2006; Rashamuse et al., 2009; Kim et al., 2010, 2014; Selvin et al., 2012).

### Sequence analysis of gene *LOB4Est*

Typical esterases of family VIII are approximately 380 residues long, with a size of 40 kDa, and show a high similarity to class C β-lactamases (Arpigny and Jaeger, 1999). Gene *LOB4Est* encodes a protein of 382 residues and 40.47 kDa, with a predicted pI of 5.58. The BLAST results showed sequence similarity with esterases and β-lactamases.

**Figure 6:**
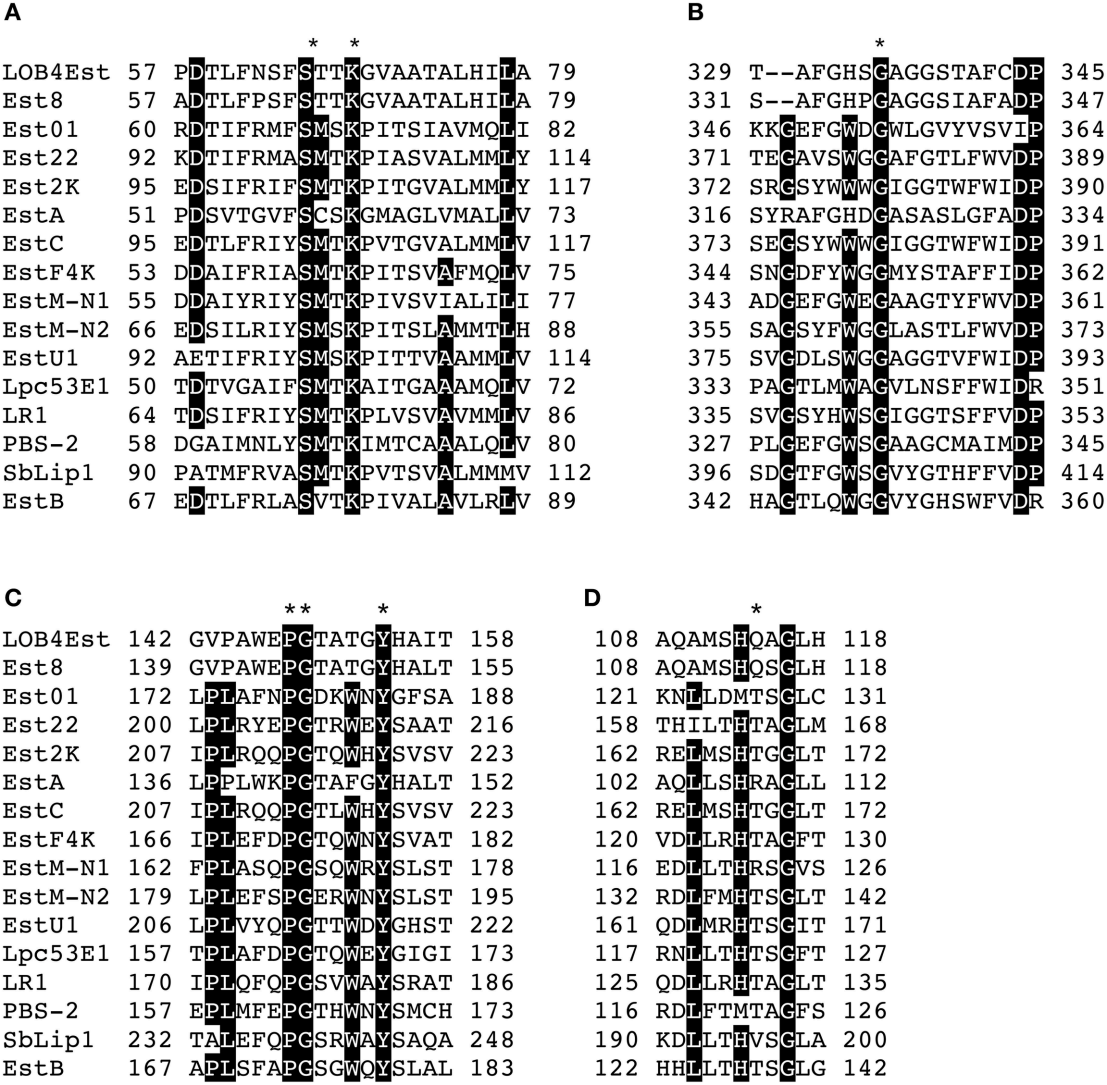
**Blocks of the multiple sequence alignment of protein LOB4Est and previously known esterases from family VIII (Arpigny and Jaeger, 1999) showing conserved motifs: (A) S-X-X-K motif, (B) G-X-S-X-G motif, (C) Y-A-N motif, (D) K-T/S-G motif, and L-L-X-H-X-X-G motif**. Black background indicate >80% identity and ^*^ indicated identity. Accession number of protein employed were AEM45116.1 for Est8 (Nacke et al., 2011), AEQ34089.1 for Est01 (Cheng et al., 2014), AGT17593.1 for Est22 (Mokoena et al., 2013), ACX51146.1 for Est2K (Kim et al., 2010), CAD61039.1 fos EstA (Schütte and Fetzner, 2007), ACH88047.1 for EstC (Rashamuse et al., 2009), AEH57832.1 for EstF4K (Ouyang et al., 2013), AEA07653.1 for EstM-N1 (Yu et al., 2011), AEA07655.1 for ESTM-N2 (Yu et al., 2011), AFU54388.1 for EstU1 (Jeon et al., 2011), AFM09717.1 for Lpc53E1 (Selvin et al., 2012), AAZ32715.1 for LR1 (Ranjan et al., 2005), AHL66978.1 for PBS-2 (Kim et al., 2014), AFK83589.1 for SbLip1 (Biver and Vandenbol, 2013), and AAF59826.1 for EstB (Wagner et al., 2002).

Multiple alignment of LOB4Est with known esterases from family VIII of lipolytic enzymes revealed the presence of several conserved motifs that are shared with β-lactamases and trans-peptidases. Most lipolytic enzymes have the catalytic serine embedded in the conserved pentapeptide GXSXG, but in LOB4Est it is located in the conserved motif S-X-X-K at positions 65–69 (Figure 6A), which is conserved in class C β-lactamases (Knox et al., 1996), penicillin binding proteins (Joris et al., 1988), and family VIII esterases (Arpigny and Jaeger, 1999). A reminiscent G-X-S-X-G motif is found in some members of this family, but it is not detected in the primary sequence of the LOB4Est protein. Alignment of LOB4Est with family VIII esterases showed that similarity is neither found with the conserved motifs G-I-S-D-G in positions 147–151 of EstB (Wagner et al., 2002) nor with G-M-S-E-G at positions 371–375 of EstC (Rashamuse et al., 2009; not shown); but weak similarity is found at positions 335–339 (G-A-G-G-S) to positions 324–328 of EstA (Figure 6B), where a putative partially conserved motif is located (Schütte and Fetzner, 2007). Several studies have proved that the Ser of this motif does not contribute to catalysis in family VIII enzymes (Petersen et al., 2001; Elend et al., 2006; Rashamuse et al., 2009; Jeon et al., 2011) but due to its location near the catalytic site it could be involved in thermostability and substrate specificity (Pérez et al., 2012).

Two highly conserved motifs of class C β-lactamases, Y-A-N, and K-T/S-G, which form the walls of the catalytic cavity (Wagner et al., 2002) and located at the middle and C- terminus, respectively, were detected in the primary sequence of LOB4Est. Only the Tyr residue at position 154, that acts as a general base catalytic group in class C β-lactamases (Schütte and Fetzner, 2007), was conserved in the Y-A-N motif (Figure 6C), and a second motif with the sequence H-S-G, whose Gly residue was conserved in every protein of the alignment, was located at positions 333-335 (Figure 6D; Wagner et al., 2002).

Moreover, a modified version of the conserved motif L-L-X-H-X-X-G (Ranjan et al., 2005) was observed in positions 110-116 (A-M-S-H-Q-A-G; Figure 6D), where the two Lys were replaced by Ala and Met, also detected in Est8 (Nacke et al., 2011).

### Cloning, biochemical characterization and heterologous expression

The gene *LOB4Es*t was cloned in the YEpFLAG-1 plasmid (Eastman Kodak Company) fused in frame to the yeast α-factor secretion signal, and expressed under the control of the yeast *ADH2* promoter and *CYC1* terminator. The product was a fusion protein with an N-terminal FLAG-tag, which allows for immunological detection and affinity purification. The recombinant plasmid was named YEpFLAG1-LOB4Est and the recombinant yeast strain transformed with this plasmid was named Sc-LOB4Est.

**Figure 7:**
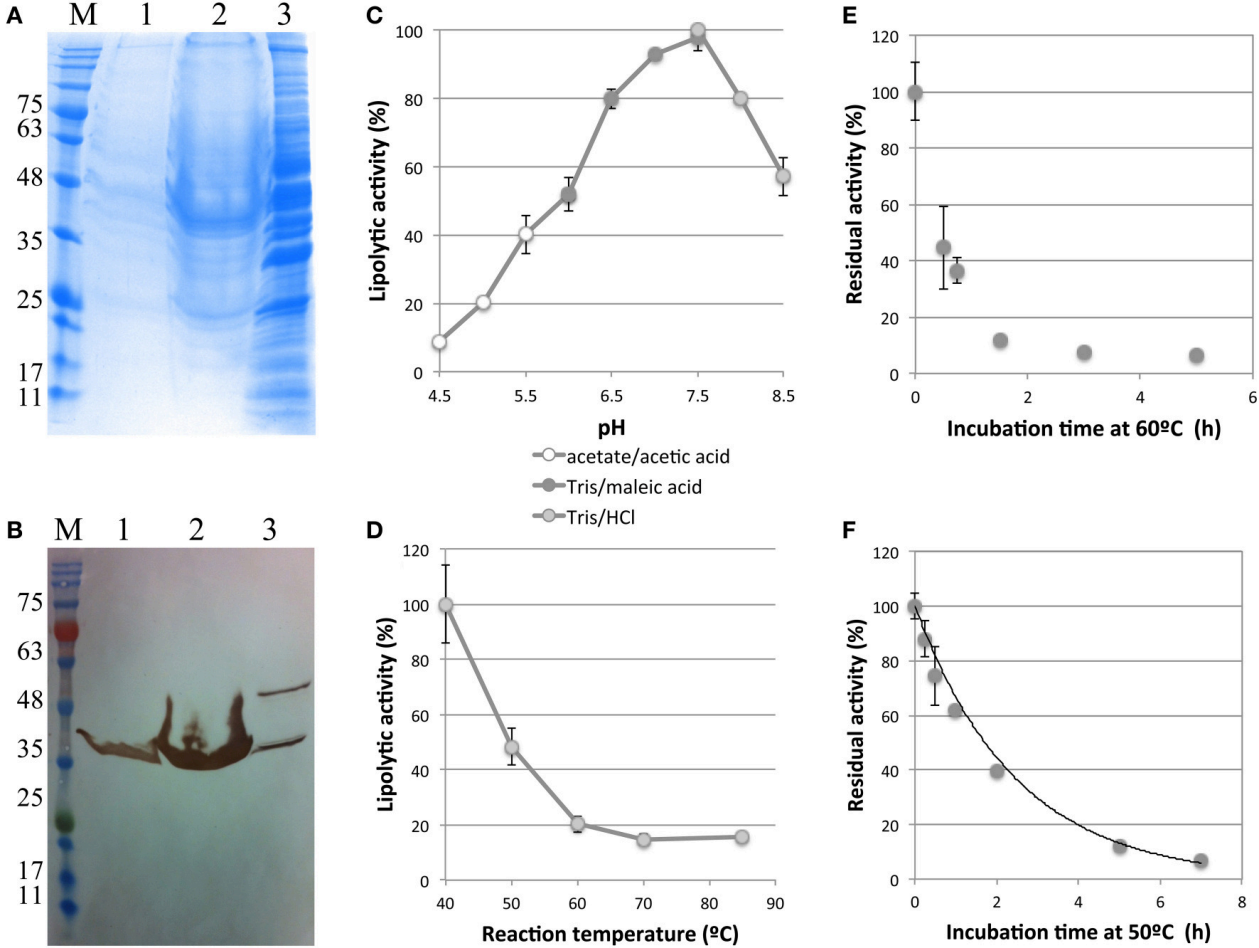
**Biochemical characterization of LOB4Est: analysis by SDS-PAGE, Coomasie stain (A), and Western Blot, anti-FLAG-M2 monoclonal antibody (B), of different fractions from a culture of Sc-LOB4Est strain: 1-supernatant, 2- 8 × concentrated supernatant, 3-crude extract**. M: NZYcolour Protein Marker II (ENZYTech). Relative activity of the recombinant enzyme at different values of pH **(C)** and temperature **(D)**. Data show mean ± standard deviation. *N* = 3. Residual activity of recombinant enzyme after incubation at 60°C **(E)** and 50°C **(F)** and first-order model of thermal deactivation at 50°C. Data show mean ± standard deviation. *N* = 3.

To verify the production and secretion of the esterase by the recombinant strain, a small culture in 2 mL of YPHSM was performed. Supernatant and crude extract were analyzed by SDS-PAGE and Western blot. A band of approximately 40 kDa was detected in both samples, in agreement with the predicted molecular mass of the recombinant protein (Figures 7A,B). A second band detected in crude extract might correspond to the pre-protein, fused with the α-factor signal sequence, which add 23 kDa to the molecular weight of the protein.

The lipolytic activity of the enzyme was studied using fresh supernatants of 2 mL cultures of the recombinant strain Sc-LOB4Est. Some biochemical characteristics of LOB4Est lipolytic activity in comparison with other family VIII lipolytic enzymes are summarized in Table S6 and discussed below.

Recombinant enzyme showed optimal activity at pH of 7.5 (Figure 7C) and 40°C (Figure 7D), in the range of tested conditions. Activity profile showed a peak at pH 7.5 but the enzyme remained highly active (above 80% of activity) in the range of pH 6.5–8, and moderately active (above 50% of activity) in the range of 6–8.5. Therefore, LOB4Est can be considered a slightly alkalophilic enzyme, although most members of this family show a stronger alkalophilic preference (Elend et al., 2006; Schütte and Fetzner, 2007; Kim et al., 2010, 2014; Takehara et al., 2012; Biver and Vandenbol, 2013). Despite of its thermophilic origin, the enzyme was more active at mesophilic temperatures and the activity dropped above 40°C, reaching 50% of maximum activity at 50°C. This is in agreement with the behavior showed in the functional screening by the fosmid clone expressing this enzyme from *E. coli*, which was the first one to appear at 40°C, and the clear halos of hydrolytic activity did not grow as much as the other clones at 65°C. For some biotechnological applications of lipolytic enzymes, such as detergent formulations, activity at moderate temperatures and basic pH is preferred when combined with enough stability.

Thermostability is an important feature of interest of thermophilic enzymes in terms of economical cost of biotransformation processes. Residual activity of the recombinant enzyme LOB4Est was measured after incubation at different time points at 60 (Figure 7E) and 50°C (Figure 7F). After 45 and 90 min of incubation at 60°C, recombinant enzyme retained 36 and 10% of initial activity, respectively, whereas after 2 h of incubation at 50°C recombinant enzyme still retained over 44 % of initial activity. Experimental data from residual activity after incubation at 50°C were adjusted to a first-order model:

(1) $$\text{LA}(\text{t})={\text{LA}}_{0}{\text{e}}^{-kt}$$

where LA(t) represents the lipolytic activity at time *t* expressed as percentage of the initial lipolytic activity (LA_0_), and *k* is the thermal deactivation constant.

Deactivation profile of the recombinant enzyme at 50°C showed a good fit to first-order model (*r*^2^ = 0.993), with a predicted half-life of 1 h and 43 min.

Microorganisms living in extreme environments subjected to high or low temperatures have adapted every component of their cellular machinery to survive under these circumstances (Ferrer et al., 2007). Thus, lipolytic proteins isolated from hot springs usually are thermostable and show high activity at the temperature according to the sample source. The protein LOB4Est, with activity at mesophilic temperatures, is one of the scarce exceptions that can be found in the literature. As examples, a lipase isolated from a hot spring in India that was thermolabile at ambient temperature (Sharma et al., 2012) and an esterase isolated from Antarctic soil that showed optimal activity at 40°C (Heath et al., 2009).

The lower optimum temperature of the recombinant enzymes compared to the living temperature of the native organisms producing them might be related to the use of a mesophilic heterologous expression system. In fact, the three enzymes mentioned in the paragraph above are of thermophilic origin but expressed in mesophilic hosts that grow at 30° (*S. cerevisiae*) or 37°C (*E. coli*). Moreover, a decrease of the optimal temperature was also shown by an esterase from *Thermus thermophilus* expressed in several yeasts and *E. coli* (López-López et al., 2010; Fuciños et al., 2011, 2014; Rocha et al., 2011); the lowest optimum temperature (40°C) was found when *S. cerevisiae* was the host, while the native *T. thermophilus* enzyme shows an optimum of temperature at 80°C. The effect of glycosylation and FLAG-tag (due to the *S. cerevisiae* expression system) on decrease of optimum temperature was discarded (López-López et al., 2010).

**Figure 8:**
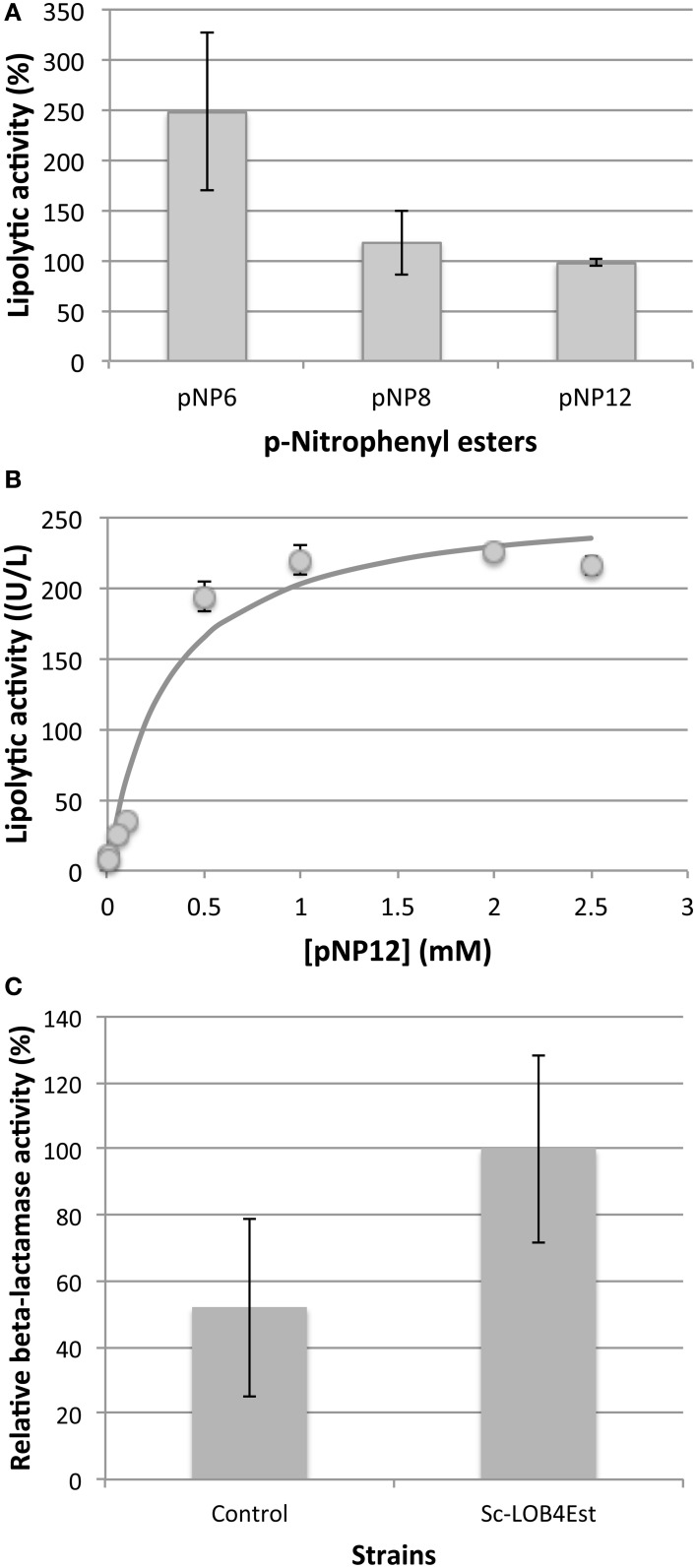
**Biochemical characterization of LOB4Est: Substrate preference (A) and Michaelis-Menten kinetics using pNP12 as substrate (B)**. Data show mean ± standart deviation. *N* = 3. **(C)** Relative β-lactamase activity of extracellular medium from cultures of Sc-LOB4Est strain and control strain. Data show mean ± standard deviation. *N* = 3.

The substrate preference was assessed using three p-Nitrophenyl esters with different side chain length: pNP6, pNP8, and pNP12. Recombinant enzyme showed obvious preference toward short-chain acyl substrates (Figure 8A), being the activity with pNP6 more than twice than the activity with pNP12, which confirms that the enzyme is indeed an esterase. Most esterases characterized from family VIII showed a marked preference toward substrates of short chain length, and only a few of them show noticeable activity using substrates with acyl chain of 10C or longer (Kim et al., 2010, 2014; Pérez et al., 2012; Selvin et al., 2012; Mokoena et al., 2013; Ouyang et al., 2013). Thus, LOB4Est is an unusual member of family VIII with a wide substrate preference.

Due to the occurrence of a certain degree of spontaneous hydrolysis of substrates with shorter side chains, pNP12 was further used for the measurements of the production and characterization of the activity of the enzyme. The initial rates of reaction of the recombinant enzyme were measured at different concentrations of pNP12 and the profile was adjusted to a Michaelis-Menten curve, which confirm the identification as an esterase (Figure 8B). Parameters of fitting are shown in Table 3.

**Table 3:** **Parametric estimations and regression coefficients of the Michaelis-Menten model and a first-order deactivation model (1) applied to the thermal deactivation of the recombinant enzyme incubated at 50°C**.

**First-order model**			**Michaelis-Menten model**		
***t* ½(h)**	***k* (h^−1^)**	***r*^2^**	**V_max_ (U/L)**	**K_m_ (mM)**	***r*^2^**
1.71	0.405	0.993	263.778	0.298	0.965

Given the sequence similarity with β-lactamase enzymes, β-lactamase activity was assayed using undiluted extracellular medium from cultures of Sc-LOB4Est strain and control strain, but although activity was slightly higher in the strain expressing the LOB4Est enzyme than in control (yeast strain transformed with empty vector), both levels of activity were very low (Figure 8C) and difference was not statistically significant (*t*-Student's test).

Most esterases from family VIII lack β-lactamase activity even though they show sequence similarity with β-lactamases. Only six members of this family have exhibited β-lactamase activity hitherto: EstA3 (Elend et al., 2006), EstC (Rashamuse et al., 2009), EstU1 (Jeon et al., 2011), EstM-N1, EstM-N2 (Kim et al., 2010; Yu et al., 2011), and PBS-2 (Kim et al., 2014). Crystallography studies suggest that the absence of β-lactamase activity in family VIII esterases is due to steric hindrance (Wagner et al., 2002). There are two 3D crystal structures available of members of the family VIII esterases, EstB and EstU1, both of which show the same arrangement of residues in the active site, but only EstU1 shows β-lactamase activity (Jeon et al., 2011). On one hand, the active site tunnel is narrower in EstB that in homologous β-lactamases with similar fold. In addition, the comparison of the structures of EstB and EstU1 allowed identifying two regions, loop Ω and R1 segment (the connecting region between the α helices α6 and α8), whose length and conformation play a critical role on the access of the substrate to the active site (Sharma et al., 2012; Cha et al., 2013). Long regions, mainly the R1 segment, block the access to the active site of voluminous substrates, thus avoiding β-lactamase activity. These regions are shorter in EstU1 than EstB. In the case of LOB4Est, while the 3D X-ray structure is not available, the architecture of the active site tunnel together with the loop Ω and R1 segment seems the most plausible hypothesis to explain the absence of β-lactamase activity.

A further investigation of the substrate preference of LOB4Est could reveal new activities as occurs with other esterases of this family.

**Figure 9:**
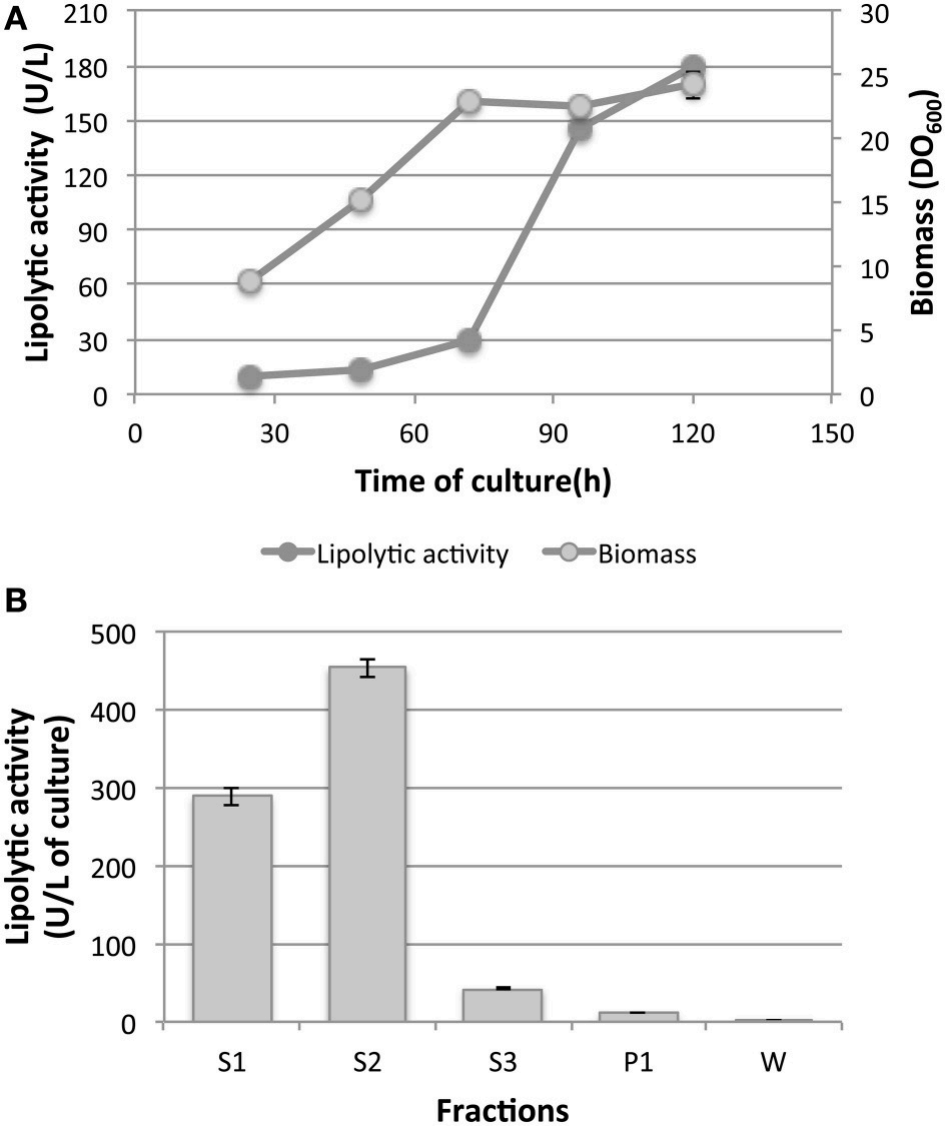
**Production of LOB4Est: (A) Biomass and extracellular lipolytic activity of a culture of the strain Sc-LOB4Est in YPHSM. (B)** Lipolytic activity in different cellular fractions of a culture of the strain Sc-LOB4Est in YPHSM. S1: extracellular, S2: periplasm, S3: cytoplasm, P1: cellular debris, W: washes. Data show mean ± standard deviation. *N* = 3.

Once the optimal reaction conditions for the lipolytic activity of LOB4Est were established, the production of the recombinant protein was analyzed. Figure 9A shows the typical profile of the biomass and extracellular lipolytic activity of a culture of the recombinant strain Sc-LOB4Est. The culture reached the stationary phase after 76 h whereas the extracellular activity showed a remarkable increase until 96 h of culture. An analysis of the lipolytic activity in the cellular fractions, using a replica-culture at this time point, revealed that 56% of the recombinant protein is secreted but still retained at the periplasmic level and a 36% is secreted to the extracellular medium (Figure 9B). This represents a higher yield than the one obtained in the expression of an esterase from *T. themophilus* HB27 using the same expression system, where only 20% of the protein was secreted to the extracellular medium (López-López et al., 2010).

## Conclusion

Metagenomic sequencing of the fosmid library constructed in this work from the thermal water of the alkaline hot spring at Lobios (Ourense, Spain) demonstrated the predominance of Bacteria over Archaea, being Deinococcus-Thermus the most abundant phylum, followed by other phyla that are also common in other thermal environments around the world.

Functional classification of the predicted ORFs revealed that the genes of the one-carbon metabolism, highlighting the serine-glyoxylate cycle, were the most abundant. It also revealed the presence of genes encoding enzymes with potential biotechnological interest, such us lipases and other hydrolases. Footprints of metabolic pathways related to the different primary nutritional groups are present in the metagenome.

Functional analysis of the metagenomic library retrieved six clones with lipolytic activity by screening on tributyrin plates. Clone FOS4, selected for further experiments, contained the gene *LOB4Est* that encodes for a novel esterase of family VIII, with sequence similarity to β-lactamases, although showing no significant β-lactamase activity; this feature was attributed to steric effects related to the architecture of the regions covering the active site, which may hinder the entrance of the β-lactamase substrate. When *p*–nitrophenyl-esters were used as substrates, *LOB4Est* showed unusually wide substrate specificity, half-life of 1 h and 43 min at 50°C, and maximal activity at 40°C and pH 7.5.

## Author contributions

OL performed the sampling, construction and functional screening of the metagenomic library, heterologous expression and characterization of the enzyme, and drafted the corresponding part of the manuscript. KK performed the bioinformatic and phylogenetic analyses, and wrote the bioinformatics section of the manuscript. MC assisted in conceiving the project and reviewing the manuscript. MG conceived the project, designed and coordinated the work, and compiled and drafted the whole manuscript. All authors contributed intellectually via scientific discussions during the work and have read and approved the final manuscript.

## Funding

The work of OL was supported by a Maria Barbeito research contract from Xunta de Galicia. KK was recruited from the European Union Seventh Framework Programme (FP7/2007-2013) under grant agreement n° 324439. The research leading to these results has received funding from project grants 10MDS373027PR (Xunta de Galicia) and GA 324439 (FP7/2007-2013). General support to the group EXPRELA was funded by Xunta de Galicia (Consolidación D.O.G. 10-10-2012. Contract Number: 2012/118) co-financed by FEDER.

### Conflict of interest statement

The authors declare that the research was conducted in the absence of any commercial or financial relationships that could be construed as a potential conflict of interest.
