# Supplementary material for: Metagenomics of an Alkaline Hot Spring in Galicia (Spain): Microbial Diversity Analysis and Screening for Novel Lipolytic Enzymes
Source: Front Microbiol. 2015 Nov 20;6:1291. doi: 10.3389/fmicb.2015.01291 (PMC4653306; doi:10.3389/fmicb.2015.01291)
Supplement: Supplementary file 3 [file DataSheet1.PDF]

Supplementary Materials

Metagenomics of an alkaline hot spring in Galicia (Spain): microbial diversity analysis and screening for novel lipolytic enzymes

Olalla López-López[1*], Kamila Knapik[1*], M. Esperanza Cerdán[1], M. Isabel González-Siso[1**]

[1] Grupo EXPRELA, Centro de Investigacións Científicas Avanzadas (CICA), Departamento de Bioloxía Celular e Molecular, Facultade de Ciencias, Universidade da Coruña, Campus de A Coruña, 15071 A Coruña, Spain

* These authors contributed equally to this work

Running title: A new family VIII esterase

Keywords: metagenomics, esterase, beta-lactamase, alkaline hot spring, biodiversity, next-generation sequencing

** Corresponding author: Dr. M. Isabel González Siso

Email: migs@udc.es

Supplementary Figures:

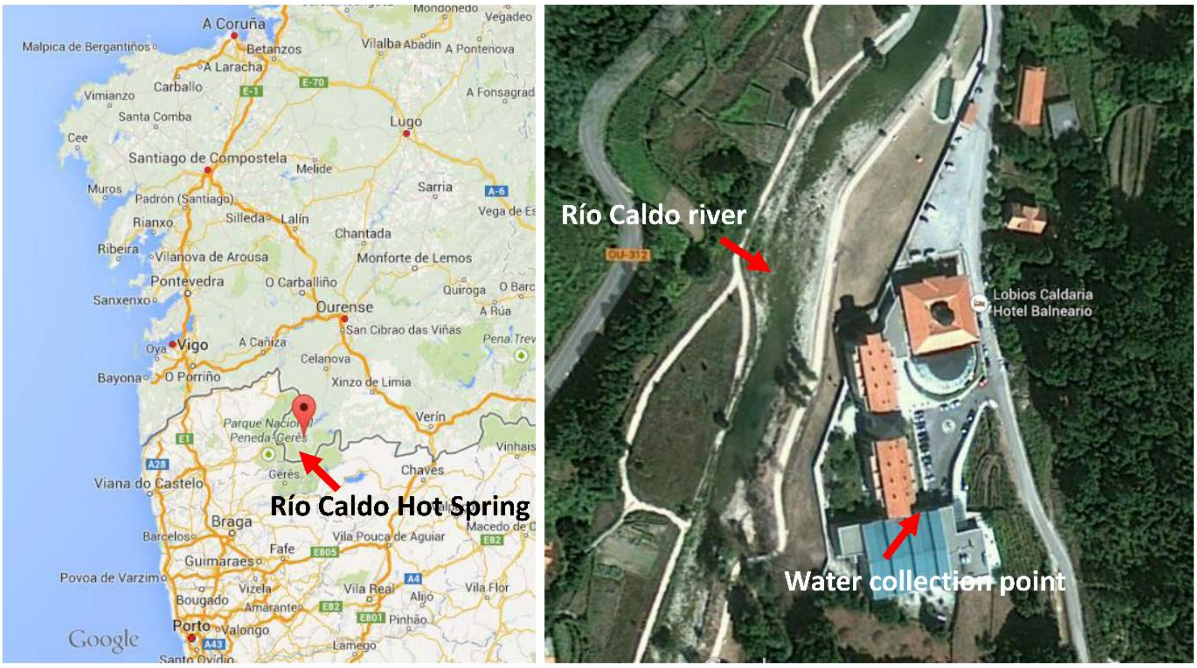

Figure S1. Sample collection site (GPS 41.86113, -8.1062).

Supplementary tables:

Table S1. Summary of sequencing results and assembly statistics.

	Parameter	Value
	**Sequencing statistics**	
**Raw reads**	No of reads	11,982,436
	Total length (bp)	1,198,243,600
	Read length (bp)	100
**Filtered reads**	No of reads	5,416,438
	Total length (bp)	541,643,800
	Read length (bp)	100
	**Assembly statistics**	
**Contigs (all)**	No of contigs	16,238
	Total length (bp)	20,375,781
	Number of contigs >20 kb	60
	Number of contigs >10 kb	215
	Number of contigs >1 kb	4625
	Number of reads in contigs	4,656,810 (86%)
	Number of singleton reads	759,628 (14%)
	Range (bp)	202-56,677
	Mean contig length (bp)	1253
	N50* contig length (bp)	2253
	Number of ORF predicted	30,057
**Contigs (≥500 bp)**	No of contigs	9,722
	Total length (bp)	17,883,789
	Number of reads in contigs	4,469,249 (82.5%)
	Range (bp)	500-56,677
	Mean contig length (bp)	1839.5
	N50* contig length (bp)	2877
	Mean GC content (%)	60.7
	Total number of unassembled data (bp)	75,962,800
	Number of ORF predicted	23,207

*****N50 contig size means that 50% of the assembled bases are contained in contigs of the N50 size or longer

Table S3. 50 most abundant species in the Lobios metagenome.

**Species**	**Metabolism**	**Oxygen requirement**	**Opt. temp. for growth (°C)**	**Opt. pH for growth**	**Phylum**	**Habitat**
Meiothermus timidus	Heterotroph	Aerobe	55-60	7.5	Deinococcus-Thermus	Thermophilic yellow-pigmented bacterium isolated from a hot spring in Portugal (Pires, Albuquerque et al. 2005)
Thermus scotoductus	Mixotroph	Facultative anaerobe	65	6.5-7.0	Deinococcus-Thermus	Sulfur-*oxidizing* thermophilic bacterium isolated from a groundwater water in a South African gold mine (Kieft, Fredrickson et al. 1999)
Ca. Caldiarchaeum subterraneum	Potential chemolithotroph	Aerobe	-	-	Thaumarchaeota	Genome reconstructed from a metagenomic library obtained from a geothermal water from subsurface gold mine in Japan. The predicted genes suggests the potential growth using hydrogen or carbon monoxide as an electron donor (Nunoura, Takaki et al. 2011)
Hydrogenobacter thermophilus	Chemolithoautotroph	Aerobe	70-75	7.2	Aquificae	Hydrogen-oxidizing thermophilic bacterium found in various geothermal sites (Pitulle, Yang et al. 1994)
Ca. Chloracidobacterium thermophilum	Chlorophototroph	Aerobe	51	7.0	Acidobacteria	*Chlorosome-containing* bacterium isolated from an alkaline siliceous hot spring in Yellowstone National Park (Bryant, Costas et al. 2007, Tank and Bryant 2015)
Thermus sp. NMX2.A1	Mixotroph	Facultative anaerobe	65	6.5-7.0	Deinococcus-Thermus	Similar to Thermus scotoductus (Kieft, Fredrickson et al. 1999)
Thermocrinis ruber	Chemolithoautotroph	Aerobe	80	7.0-8.5	Aquificae	Hyperthermophilic bacterium isolated from the alkaline Octopus Spring in Yellowstone National Park. Able to grow chemolithoautotrophically with hydrogen, thiosulfate, and elemental sulfur as electron donors (Huber, Eder et al. 1998)
Ca. Nitrososphaera gargensis	Chemolithoautotroph	Aerobe	46	7.9	Thaumarchaeota	Moderately thermophilic ammonia-oxidizing bacterium isolated from an alkaline hot spring in Russia (Lebedeva, Alawi et al. 2005, Hatzenpichler, Lebedeva et al. 2008)
Rhodothermus marinus	Heterotroph	Aerobe	65	7.0	Bacteroidetes	Moderately halophilic bacterium isolated from a submarine alkaline hot spring in Iceland (Alfredsson GA 1988)
Gemmata obscuriglobus	Heterotroph	Aerobe	28	7.0	Planctomycetes	Freshwater bacterium (Fuerst and Webb 1991)
Ca. Nitrospira defluvii	Chemolithoautotroph	Aerobe	28	-	Nitrospirae	Nitrite-oxidizing bacterium isolated from an alkaline

						activated sludge (Spieck, Hartwig et al. 2006)
Meiothermus silvanus	Heterotroph	Aerobe	55	8.0-8.5	Deinococcus-Thermus	Thermophilic orange-red-pigmented bacterium isolated from an alkaline hot spring in Portugal (Tenreiro, Nobre et al. 1995)
Thermocrinis albus	Chemolithoautotroph	Aerobe	88	7.5	Aquificae	Hyperthermophilic, sulfur- *and* hydrogen-oxidizing bacterium isolated from whitish streamers in Iceland (Eder and Huber 2002)
Ca. Solibacter usitatus	Chemoorganotroph	Aerobe	30	3.5-6.5	Acidobacteria	Isolated from soil in Australia (Ward, Challacombe et al. 2009)
Thermus igniterrae	Heterotroph	Aerobe	65	7.5-8.5	Deinococcus-Thermus	Isolated from a hot spring in Iceland (Chung, Rainey et al. 2000)
Caldilinea aerophila	Chemoorganotroph	Facultative aerobe	55	7.5-8.0	Chloroflexi	Thermophile isolated from a hot spring in Japan (Sekiguchi, Yamada et al. 2003)
Anaerolinea thermophila	Chemoorganotroph	Anaerobe	55	7.0	Chloroflexi	Thermophilic bacterium isolated from a sludge reactor (Sekiguchi, Yamada et al. 2003)
Thermomicrobium roseum	Chemoheterotroph	Obligate aerobe	70-75	8.2–8.5	Chloroflexi	Thermophilic CO-Oxidizing bacterium isolated from an alkaline siliceous hot spring in Yellowstone National Park (Wu, Raymond et al. 2009)
Thermus islandicus	Mixotroph	Aerobe	65	6.0-7.0	Deinococcus-Thermus	Sulfur-oxidizing bacterium isolated from Icelandic hot spring (Bjornsdottir, Petursdottir et al. 2009)
Ignavibacterium album	Chemoheterotroph	Anaerobe	45	7.0-7.5	Ignavibacteriae	Isolated from a hot spring in Japan (Iino, Mori et al. 2010)
Sphaerobacter thermophilus	Chemoheterotroph	Obligate aerobe	55	8.5	Chloroflexi	Isolated from an aerated laboratory-scale fermenter receiving municipal sewage sludge (Pati, Labutti et al. 2010)
Chthonomonas calidirosea	Chemoheterotroph	Aerobe	68	5.3	Armatimonadetes	Thermophilic bacterium isolated from geothermally heated soil at in New Zealand (Lee, Dunfield et al. 2011)
Thermodesulfovibrio yellowstonii	-	Anaerobe	65	-	Nitrospirae	Thermophilic sulfate-reducing bacterium isolated from thermal vent water in Yellowstone Lake in USA (Henry, Devereux et al. 1994)
Ca. Koribacter versatilis	Chemoorganotroph	Aerobe	30	4.0-6.5	Acidobacteria	Isolated from soil in Australia (Ward, Challacombe et al. 2009)
Ca. Methylomirabilis oxyfera	Methanotroph	Anaerobe	25-30	7.0-8.0	unclassified Bacteria	Methane-oxidizing and nitrite-reducing bacterium isolated from sediment (Ettwig, Butler et al. 2010)
Meiothermus ruber	Heterotroph	Obligate aerobe	60	7.0	Deinococcus-Thermus	Isolated from a hot spring in Kamchatka (Russia) (Loginova LG 1984)

Roseiflexus sp. RS-1	Photoheterotroph	Aerobe	55-60	8.1	Chloroflexi	Isolated from an alkaline siliceous hot spring in Yellowstone National Park (van der Meer, Klatt et al. 2010)
Methylococcus capsulatus	Methanotroph	Aerobe	45	6.8	Proteobacteria	Thermotolerant methane-oxidizing bacterium isolated from a hot spring (Whittenbury, Phillips et al. 1970, Ward, Larsen et al. 2004)
Melioribacter roseus	Chemoorganotroph	Facultative anaerobe	52-55	7.5	Ignavibacteriae	Thermophilic obligate organotroph isolated from a hot spring microbial mat from Russia (Podosokorskaya, Kadnikov et al. 2013)
Roseiflexus castenholzii	Photoheterotroph, chemoheterotroph	Facultative anaerobe	50	7.5-8.0	Chloroflexi	Thermophilic, photosynthetic bacterium isolated from a bacterial mat in a Japanese hot spring (Hanada, Takaichi et al. 2002)
Ktedonobacter racemifer	Heterotroph	Aerobe	28-33	6	Chloroflexi	Mesophilic bacterium isolated from an Italian soil (Cavaletti, Monciardini et al. 2006)
Thermaerobacter subterraneus	Heterotroph	Aerobe	70	8.5	Firmicutes	Thermophilic bacterium isolated from a sample collected from an open drain run-off channel of a bore in the Great Artesian Basin of Australia (Spanevello, Yamamoto et al. 2002)
Thermus thermophilus	Heterotroph	Aerobe	65	7.5	Deinococcus-Thermus	Thermophilic, halotolerant bacterium isolated from a Japanese hot spring (K 1974)
Zavarzinella formosa	Chemoheterotroph	Aerobe	20-25	5.5-6.0	Planctomycetes	Mesophilic bacterium isolated from acidic wetlands (Kulichevskaya, Baulina et al. 2009)
Singulisphaera acidiphila	Chemoheterotroph	Aerobe	20-26	5.0-6.2	Planctomycetes	Mesophilic bacterium isolated from acidic wetlands (Kulichevskaya, Ivanova et al. 2008)
Hydrogenivirga sp.128-5-R1-1	Chemolithoautotroph	Microaerophile	70	6.0	Aquificae	Hydrogen- and sulfur -oxidizing bacterium isolated from a hydrothermal vent in the Pacific Ocean at a depth of 2200 meters (Freedman, Zhu et al. 2012)
Aquifex aeolicus	Chemolithoautotroph	Microaerophile	85	-	Aquificae	Extremely thermophilic, hydrogen-oxidizing bacterium isolated from hot spring environment (Deckert, Warren et al. 1998)
Marinithermus hydrothermalis	Heterotroph	Obligate aerobe	67.5	7.0	Deinococcus-Thermus	Thermophilic marine bacterium isolated from a deep-sea hydrothermal vent in Japan (Sako, Nakagawa et al. 2003)
Thermaerobacter marianensis	Heterotroph	Obligate aerobe	74-76	7.0-7.5	Firmicutes	Moderately thermophilic methylotrophic sulfate-reducing bacterium isolated from deep subsurface thermal mineral water (Visser, Worm et al. 2013)

Desulfotomaculum kuznetsovii	Methylotroph, autotroph	Obligate anaerobe	60		Firmicutes	Thermophilic bacterium found in deep subsurface thermal mineral water at a depth of about 3,000 m (Visser, Worm et al. 2013)
Methylohalobius crimeensis	Methanotroph	Aerobe	30	6.5-7.5		Moderately halophilic, methanotrophic bacterium isolated from hypersaline lake in Ukraine (Heyer, Berger et al. 2005)
Nitrolancea hollandica	Chemolithoautotroph	Obligate aerobe	40	6.8-7.5		Nitrite-oxidizing bacterium isolated from a nitrifying bioreactor (Sorokin, Vejmelkova et al. 2014)
Ammonifex degensii	Facultative chemolithoautotroph	Anaerobe	70	7.5		Extremely thermophilic hydrogen-oxidizing, nitrate reducing bacterium isolated from a neutral volcanic hot spring (Huber, Rossnagel et al. 1996)
Ca. Poribacteria sp. WGA-4E	-	-	-	-		Marine sponge symbiotic bacterium (Kamke, Sczyrba et al. 2013)
Dehalococcoides mccartyi	Chemotroph	Anaerobe	25-30	6.5-8.0		Organohalide-respiring bacterium isolated anoxic digester sludge (Loffler, Yan et al. 2013)
Thermosediminibacter oceani	Chemoorganotroph	Anaerobe	68	7.5		Thermophilic bacterium isolated from a deep sea sediment of Pacific ocean (Lee, Wagner et al. 2005)
Desulfomonile tiedjei	Autotroph, chemoorganotroph	Anaerobe	20-38	6.5-7.8		Dehalogenating, sulfate-reducing bacterium isolated from municipal digester sludge (DeWeerd K. 1990)
Ca. Acetothermus autotrophicum	Chemolithoautotroph	-	Predicted 85	-		Genome reconstructed from a subsurface thermophilic microbial mat community. Encodes genes for acetogenesis and methanogenesis pathways (Takami, Noguchi et al. 2012)
Symbiobacterium thermophilum	-	Microaerophile	45-65	7.5		Isolated from compost (Ohno, Shiratori et al. 2000)
Sorangium cellulosum	-	Aerobe	30	7-10	Proteobacteria	Mesophilic bacterium isolated from alkaline soils near an alkaline lake (Han, Li et al. 2013)

Table S5. Summary of BLASTP results against lipase database.

**ORF ID**	**Gene name**	**Organism name**	**NCBI accession number**	**Identity (%)**	**Query coverage (%)**	**E-value**
6253	lipase/esterase	uncultured bacterium	AAX37299	88	98	0
19628	carboxylesterase	*Meiothermus timidus*	WP_018465292	86	100	2e-133
15491	carboxylesterase	*Meiothermus chliarophilus*	WP_027891785	93	86	2e-59
17667	carboxylesterase	*Meiothermus ruber*	WP_013013008	77	100	6e-40
12745	carboxylesterase	*Thermus scotoductus*	WP_041438818	100	100	0
13704	G-D-S-L family lipolytic protein	*Meiothermus chliarophilus*	WP_027892767	85	100	4e-125
13594	lipase/esterase	uncultured marine crenarchaeote E37-7F	ADQ54403	59	99	2e-91
19149	lipase/esterase	*Clostridium ultunense*	WP_005588344	38	97	2e-53
2073	phospholipase	*Hydrogenivirga sp.* 128-5-R1-1	WP_008286792	59	100	9e-103
6726	phospholipase	*Thermus scotoductus*	WP_019551729	97	100	2e-121
8181	serine esterase	*Bdellovibrio bacteriovorus W*	AHI06854	32	29	3e-09

Table S6: Biochemical features of lipolytic enzymes in family VIII.

Protein Name	Source	Optimal pH	Optimal Tª	Thermal stability	Substrate preference*	Reference
PBS-2 esterase	*Paenibacillus* sp. PBS-2	9	30ºC	RA after 30 min-50ºC: >80 %	C2 to C8	(Kim et al., 2014)
Est01	Metagenomic: biogas digester	8	20ºC	RA after 1 h-40ºC: 20 %	C2 to C8	(Cheng et al., 2014)
SBLip1	Metagenomic: forest soil	10	35ºC	NT	C2 to C4	(Biver and Vandenbol, 2013)
Est22	Metagenomic: leachate	8	30ºC	RA after 30 min-50ºC: 50 %	C3 to C10	(Mokoena et al., 2013)
EstF4K	Metagenomic: soil	8	50ºC	RA after 5 h-50ºC: 60 %	C3 to C6	(Ouyang et al., 2013)
Lpc53E1	Metagenomic: marine sponge	7	40ºC	RA after 1 h-90ºC: 57.6 %	C12 to C16	(Selvin et al., 2012)
EstAC	*Sporosarcina* sp.	9	40ºC	RA after 1 h-45ºC: 55 %	NT	(Takehara et al., 2012)
EstM-N1	Metagenomic: arctic soil	9	20ºC	Extremely thermolabile over 40ºC	C4	(Yu et al., 2011)
EstM-N2		9	30ºC		C4	
EstU1	Metagenomic: soil	8.5	45ºC	NT	C2 to C10	(Jeon et al., 2011)
Est08	Metagenomic: soil	NT	NT	NT	C4 to C8	(Nacke et al., 2011)
Est2K	Metagenomic: compost	10	50ºC	RA after 15 min-40ºC: 68 %	C4 to C10	(Kim et al., 2010)
EstC	Metagenomic: leachate	NT	40ºC	RA after 30 min-50ºC: 50 %	C2 to C8	(Rashamuse et al., 2009)
EstA	*Arthrobacter nitroguajacolicus* Rü61a	9.5	50-60ºC	RA after 19 days-50ºC: 53 %	NT	(Schütte and Fetzner, 2007)
EstA3	Metagenomic: drinking water	9	50ºC	Stable at 40ºC for 60 min.	C4 to C6	(Elend et al., 2006)
EstCE1	Metagenomic: soil	10	47ºC		C4 to C6	
EstB	*Burkholderia gladioli*	7	43ºC	RA after 20 min-40ºC: 50 %	C2 to C5	(Petersen et al., 2001)
LOB4Est	Metagenomic: thermal water	7.5	40ºC	RA after 2 h-50ºC: 44 %	C6	This work

RA: residual activity.
NT: not tested.
* side-chain lenght of pNP esters in carbon atoms.

References Table S3

Alfredsson GA, K. J., Hjörleifsdottir S and Stetter KO (1988). "Rhodothermus marinus, gen. nov., sp. nov., a Thermophilic, Halophilic Bacterium from Submarine Hot Springs in Iceland." Microbiology **134**(2): 299-306.

Bjornsdottir, S. H., S. K. Petursdottir, G. O. Hreggvidsson, S. Skirnisdottir, S. Hjorleifsdottir, J. Arnfinnsson and J. K. Kristjansson (2009). "Thermus islandicus sp. nov., a mixotrophic sulfur-oxidizing bacterium isolated from the Torfajokull geothermal area." Int J Syst Evol Microbiol **59**(Pt 12): 2962-2966.

Bryant, D. A., A. M. Costas, J. A. Maresca, A. G. Chew, C. G. Klatt, M. M. Bateson, L. J. Tallon, J. Hostetler, W. C. Nelson, J. F. Heidelberg and D. M. Ward (2007). "Candidatus Chloracidobacterium thermophilum: an aerobic phototrophic Acidobacterium." Science **317**(5837): 523-526.

Cavaletti, L., P. Monciardini, R. Bamonte, P. Schumann, M. Rohde, M. Sosio and S. Donadio (2006). "New lineage of filamentous, spore-forming, gram-positive bacteria from soil." Appl Environ Microbiol **72**(6): 4360-4369.

Chung, A. P., F. A. Rainey, M. Valente, M. F. Nobre and M. S. da Costa (2000). "Thermus igniterrae sp. nov. and Thermus antranikianii sp. nov., two new species from Iceland." Int J Syst Evol Microbiol **50 Pt 1**: 209-217.

Deckert, G., P. V. Warren, T. Gaasterland, W. G. Young, A. L. Lenox, D. E. Graham, R. Overbeek, M. A. Snead, M. Keller, M. Aujay, R. Huber, R. A. Feldman, J. M. Short, G. J. Olsen and R. V. Swanson (1998). "The complete genome of the hyperthermophilic bacterium Aquifex aeolicus." Nature **392**(6674): 353-358.

DeWeerd K., M. L., Tanner R., Woese C., Suflita J (1990). "Desulfomonile tiedjei gen. nov. and sp. nov., a novel anaerobic, dehalogenating, sulfate-reducing bacterium." Archives of Microbiology **154**: 23-30.

Eder, W. and R. Huber (2002). "New isolates and physiological properties of the Aquificales and description of Thermocrinis albus sp. nov." Extremophiles **6**(4): 309-318.

Ettwig, K. F., M. K. Butler, D. Le Paslier, E. Pelletier, S. Mangenot, M. M. Kuypers, F. Schreiber, B. E. Dutilh, J. Zedelius, D. de Beer, J. Gloerich, H. J. Wessels, T. van Alen, F. Luesken, M. L. Wu, K. T. van de Pas-Schoonen, H. J. Op den Camp, E. M. Janssen-Megens, K. J. Francoijs, H. Stunnenberg, J. Weissenbach, M. S. Jetten and M. Strous (2010). "Nitrite-driven anaerobic methane oxidation by oxygenic bacteria." Nature **464**(7288): 543-548.

Freedman, Z., C. Zhu and T. Barkay (2012). "Mercury resistance and mercuric reductase activities and expression among chemotrophic thermophilic Aquificae." Appl Environ Microbiol **78**(18): 6568-6575.

Fuerst, J. A. and R. I. Webb (1991). "Membrane-bounded nucleoid in the eubacterium Gemmata obscuriglobus." Proc Natl Acad Sci U S A **88**(18): 8184-8188.

Han, K., Z. F. Li, R. Peng, L. P. Zhu, T. Zhou, L. G. Wang, S. G. Li, X. B. Zhang, W. Hu, Z. H. Wu, N. Qin and Y. Z. Li (2013). "Extraordinary expansion of a Sorangium cellulosum genome from an alkaline milieu." Sci Rep **3**: 2101.

Hanada, S., S. Takaichi, K. Matsuura and K. Nakamura (2002). "Roseiflexus castenholzii gen. nov., sp. nov., a thermophilic, filamentous, photosynthetic bacterium that lacks chlorosomes." Int J Syst Evol Microbiol **52**(Pt 1): 187-193.

Hatzenpichler, R., E. V. Lebedeva, E. Spieck, K. Stoecker, A. Richter, H. Daims and M. Wagner (2008). "A moderately thermophilic ammonia-oxidizing crenarchaeote from a hot spring." Proc Natl Acad Sci U S A **105**(6): 2134-2139.

Henry, E. A., R. Devereux, J. S. Maki, C. C. Gilmour, C. R. Woese, L. Mandelco, R. Schauder, C. C. Remsen and R. Mitchell (1994). "Characterization of a new thermophilic sulfate-reducing bacterium Thermodesulfovibrio yellowstonii, gen. nov. and sp. nov.: its phylogenetic relationship to Thermodesulfobacterium commune and their origins deep within the bacterial domain." Arch Microbiol **161**(1): 62-69.

Heyer, J., U. Berger, M. Hardt and P. F. Dunfield (2005). "Methylohalobius crimeensis gen. nov., sp. nov., a moderately halophilic, methanotrophic bacterium isolated from hypersaline lakes of Crimea." Int J Syst Evol Microbiol **55**(Pt 5): 1817-1826.

Huber, R., W. Eder, S. Heldwein, G. Wanner, H. Huber, R. Rachel and K. O. Stetter (1998). "Thermocrinis ruber gen. nov., sp. nov., A pink-filament-forming hyperthermophilic bacterium isolated from yellowstone national park." Appl Environ Microbiol **64**(10): 3576-3583.

Huber, R., P. Rossnagel, C. R. Woese, R. Rachel, T. A. Langworthy and K. O. Stetter (1996). "Formation of ammonium from nitrate during chemolithoautotrophic growth of the extremely thermophilic bacterium ammonifex degensii gen. nov. sp. nov." Syst Appl Microbiol **19**(1): 40-49.

Iino, T., K. Mori, Y. Uchino, T. Nakagawa, S. Harayama and K. Suzuki (2010). "Ignavibacterium album gen. nov., sp. nov., a moderately thermophilic anaerobic bacterium isolated from microbial mats at a terrestrial hot spring and proposal of Ignavibacteria classis nov., for a novel lineage at the periphery of green sulfur bacteria." Int J Syst Evol Microbiol **60**(Pt 6): 1376-1382.

K, O. T. a. I. (1974). "Description of Thermus thermophilus (Yoshida and Oshima) comb. nov., a Nonsporulating Thermophilic Bacterium from a Japanese Thermal Spa." IJSEM **24**(1): 102-112.

Kamke, J., A. Sczyrba, N. Ivanova, P. Schwientek, C. Rinke, K. Mavromatis, T. Woyke and U. Hentschel (2013). "Single-cell genomics reveals complex carbohydrate degradation patterns in poribacterial symbionts of marine sponges." ISME J **7**(12): 2287-2300.

Kieft, T. L., J. K. Fredrickson, T. C. Onstott, Y. A. Gorby, H. M. Kostandarithes, T. J. Bailey, D. W. Kennedy, S. W. Li, A. E. Plymale, C. M. Spadoni and M. S. Gray (1999). "Dissimilatory reduction of Fe(III) and other electron acceptors by a Thermus isolate." Appl Environ Microbiol **65**(3): 1214-1221.

Kulichevskaya, I. S., O. I. Baulina, P. L. Bodelier, W. I. Rijpstra, J. S. Damste and S. N. Dedysh (2009). "Zavarzinella formosa gen. nov., sp. nov., a novel stalked, Gemmata-like planctomycete from a Siberian peat bog." Int J Syst Evol Microbiol **59**(Pt 2): 357-364.

Kulichevskaya, I. S., A. O. Ivanova, O. I. Baulina, P. L. Bodelier, J. S. Damste and S. N. Dedysh (2008). "Singulisphaera acidiphila gen. nov., sp. nov., a non-filamentous, Isosphaera-like planctomycete from acidic northern wetlands." Int J Syst Evol Microbiol **58**(Pt 5): 1186-1193.

Lebedeva, E. V., M. Alawi, C. Fiencke, B. Namsaraev, E. Bock and E. Spieck (2005). "Moderately thermophilic nitrifying bacteria from a hot spring of the Baikal rift zone." FEMS Microbiol Ecol **54**(2): 297-306.

Lee, K. C., P. F. Dunfield, X. C. Morgan, M. A. Crowe, K. M. Houghton, M. Vyssotski, J. L. Ryan, K. Lagutin, I. R. McDonald and M. B. Stott (2011). "Chthonomonas calidirosea gen. nov., sp. nov., an aerobic, pigmented, thermophilic micro-organism of a novel bacterial class, Chthonomonadetes classis nov., of the newly described phylum Armatimonadetes originally designated candidate division OP10." Int J Syst Evol Microbiol **61**(Pt 10): 2482-2490.

Lee, Y. J., I. D. Wagner, M. E. Brice, V. V. Kevbrin, G. L. Mills, C. S. Romanek and J. Wiegel (2005). "Thermosediminibacter oceani gen. nov., sp. nov. and Thermosediminibacter litoriperuensis sp. nov., new anaerobic thermophilic bacteria isolated from Peru Margin." Extremophiles **9**(5): 375-383.

Loffler, F. E., J. Yan, K. M. Ritalahti, L. Adrian, E. A. Edwards, K. T. Konstantinidis, J. A. Muller, H. Fullerton, S. H. Zinder and A. M. Spormann (2013). "Dehalococcoides mccartyi gen. nov., sp. nov., obligately organohalide-respiring anaerobic bacteria relevant to halogen cycling and bioremediation, belong to a novel bacterial class, Dehalococcoidia classis nov., order Dehalococcoidales ord. nov. and family Dehalococcoidaceae fam. nov., within the phylum Chloroflexi." Int J Syst Evol Microbiol **63**(Pt 2): 625-635.

Loginova LG, E. L., Golovacheva RS and Seregina LM (1984). "Therrnus ruber sp. nov., norn. rev. ." International Journal Of Systematic Bacteriology **34**(4): 498-499

Nunoura, T., Y. Takaki, J. Kakuta, S. Nishi, J. Sugahara, H. Kazama, G. J. Chee, M. Hattori, A. Kanai, H. Atomi, K. Takai and H. Takami (2011). "Insights into the evolution of Archaea and eukaryotic protein modifier systems revealed by the genome of a novel archaeal group." Nucleic Acids Res **39**(8): 3204-3223.

Ohno, M., H. Shiratori, M. J. Park, Y. Saitoh, Y. Kumon, N. Yamashita, A. Hirata, H. Nishida, K. Ueda and T. Beppu (2000). "Symbiobacterium thermophilum gen. nov., sp. nov., a symbiotic thermophile that depends on co-culture with a Bacillus strain for growth." Int J Syst Evol Microbiol **50 Pt 5**: 1829-1832.

Pati, A., K. Labutti, R. Pukall, M. Nolan, T. Glavina Del Rio, H. Tice, J. F. Cheng, S. Lucas, F. Chen, A. Copeland, N. Ivanova, K. Mavromatis, N. Mikhailova, S. Pitluck, D. Bruce, L. Goodwin, M. Land, L. Hauser, Y. J. Chang, C. D. Jeffries, A. Chen, K. Palaniappan, P. Chain, T. Brettin, J. Sikorski, M. Rohde, M. Goker, J. Bristow, J. A. Eisen, V. Markowitz, P. Hugenholtz, N. C. Kyrpides, H. P. Klenk and A. Lapidus (2010). "Complete genome sequence of Sphaerobacter thermophilus type strain (S 6022)." Stand Genomic Sci **2**(1): 49-56.

Pires, A. L., L. Albuquerque, I. Tiago, M. F. Nobre, N. Empadinhas, A. Verissimo and M. S. da Costa (2005). "Meiothermus timidus sp. nov., a new slightly thermophilic yellow-pigmented species." FEMS Microbiol Lett **245**(1): 39-45.

Pitulle, C., Y. Yang, M. Marchiani, E. R. Moore, J. L. Siefert, M. Aragno, P. Jurtshuk, Jr. and G. E. Fox (1994). "Phylogenetic position of the genus Hydrogenobacter." Int J Syst Bacteriol **44**(4): 620-626.

Podosokorskaya, O. A., V. V. Kadnikov, S. N. Gavrilov, A. V. Mardanov, A. Y. Merkel, O. V. Karnachuk, N. V. Ravin, E. A. Bonch-Osmolovskaya and I. V. Kublanov (2013). "Characterization of Melioribacter roseus gen. nov., sp. nov., a novel facultatively anaerobic thermophilic cellulolytic bacterium from the class Ignavibacteria, and a proposal of a novel bacterial phylum Ignavibacteriae." Environ Microbiol **15**(6): 1759-1771.

Sako, Y., S. Nakagawa, K. Takai and K. Horikoshi (2003). "Marinithermus hydrothermalis gen. nov., sp. nov., a strictly aerobic, thermophilic bacterium from a deep-sea hydrothermal vent chimney." Int J Syst Evol Microbiol **53**(Pt 1): 59-65.

Sekiguchi, Y., T. Yamada, S. Hanada, A. Ohashi, H. Harada and Y. Kamagata (2003). "Anaerolinea thermophila gen. nov., sp. nov. and Caldilinea aerophila gen. nov., sp. nov., novel filamentous thermophiles that represent a previously uncultured lineage of the domain Bacteria at the subphylum level." Int J Syst Evol Microbiol **53**(Pt 6): 1843-1851.

Sorokin, D. Y., D. Vejmelkova, S. Lucker, G. M. Streshinskaya, W. I. Rijpstra, J. S. Sinninghe Damste, R. Kleerbezem, M. van Loosdrecht, G. Muyzer and H. Daims (2014). "Nitrolancea hollandica gen. nov., sp. nov., a chemolithoautotrophic nitrite-oxidizing bacterium isolated from a bioreactor belonging to the phylum Chloroflexi." Int J Syst Evol Microbiol **64**(Pt 6): 1859-1865.

Spanevello, M. D., H. Yamamoto and B. K. Patel (2002). "Thermaerobacter subterraneus sp. nov., a novel aerobic bacterium from the Great Artesian Basin of Australia, and emendation of the genus Thermaerobacter." Int J Syst Evol Microbiol **52**(Pt 3): 795-800.

Spieck, E., C. Hartwig, I. McCormack, F. Maixner, M. Wagner, A. Lipski and H. Daims (2006). "Selective enrichment and molecular characterization of a previously uncultured Nitrospira-like bacterium from activated sludge." Environ Microbiol **8**(3): 405-415.

Takami, H., H. Noguchi, Y. Takaki, I. Uchiyama, A. Toyoda, S. Nishi, G. J. Chee, W. Arai, T. Nunoura, T. Itoh, M. Hattori and K. Takai (2012). "A deeply branching thermophilic bacterium with an ancient acetyl-CoA pathway dominates a subsurface ecosystem." PLoS One **7**(1): e30559.

Tank, M. and D. A. Bryant (2015). "Nutrient requirements and growth physiology of the photoheterotrophic Acidobacterium, Chloracidobacterium thermophilum." Front Microbiol **6**: 226.

Tenreiro, S., M. F. Nobre and M. S. da Costa (1995). "Thermus silvanus sp. nov. and Thermus chliarophilus sp. nov., two new species related to thermus ruber but with lower growth temperatures." Int J Syst Bacteriol **45**(4): 633-639.

van der Meer, M. T., C. G. Klatt, J. Wood, D. A. Bryant, M. M. Bateson, L. Lammerts, S. Schouten, J. S. Damste, M. T. Madigan and D. M. Ward (2010). "Cultivation and genomic, nutritional, and lipid biomarker characterization of Roseiflexus strains closely related to predominant in situ populations inhabiting Yellowstone hot spring microbial mats." J Bacteriol **192**(12): 3033-3042.

Visser, M., P. Worm, G. Muyzer, I. A. Pereira, P. J. Schaap, C. M. Plugge, J. Kuever, S. N. Parshina, T. N. Nazina, A. E. Ivanova, R. Bernier-Latmani, L. A. Goodwin, N. C. Kyrpides, T. Woyke, P. Chain, K. W. Davenport, S. Spring, H. P. Klenk and A. J. Stams (2013). "Genome analysis of Desulfotomaculum kuznetsovii strain 17(T) reveals a physiological similarity with Pelotomaculum thermopropionicum strain SI(T)." Stand Genomic Sci **8**(1): 69-87.

Ward, N., O. Larsen, J. Sakwa, L. Bruseth, H. Khouri, A. S. Durkin, G. Dimitrov, L. Jiang, D. Scanlan, K. H. Kang, M. Lewis, K. E. Nelson, B. Methe, M. Wu, J. F. Heidelberg, I. T. Paulsen, D. Fouts, J. Ravel, H. Tettelin, Q. Ren, T. Read, R. T. DeBoy, R. Seshadri, S. L. Salzberg, H. B. Jensen, N. K. Birkeland, W. C. Nelson, R. J. Dodson, S. H. Grindhaug, I. Holt, I. Eidhammer, I. Jonasen, S. Vanaken, T. Utterback, T. V. Feldblyum, C. M. Fraser, J. R. Lillehaug and J. A. Eisen (2004). "Genomic insights into methanotrophy: the complete genome sequence of Methylococcus capsulatus (Bath)." PLoS Biol **2**(10): e303.

Ward, N. L., J. F. Challacombe, P. H. Janssen, B. Henrissat, P. M. Coutinho, M. Wu, G. Xie, D. H. Haft, M. Sait, J. Badger, R. D. Barabote, B. Bradley, T. S. Brettin, L. M. Brinkac, D. Bruce, T. Creasy, S. C. Daugherty, T. M. Davidsen, R. T. DeBoy, J. C. Detter, R. J. Dodson, A. S. Durkin, A. Ganapathy, M. Gwinn-Giglio, C. S. Han, H. Khouri, H. Kiss, S. P. Kothari, R. Madupu, K. E. Nelson, W. C. Nelson, I. Paulsen, K. Penn, Q. Ren, M. J. Rosovitz, J. D. Selengut, S. Shrivastava, S. A. Sullivan, R. Tapia, L. S. Thompson, K. L. Watkins, Q. Yang, C. Yu, N. Zafar, L. Zhou and C. R. Kuske (2009). "Three genomes from the phylum Acidobacteria provide insight into the lifestyles of these microorganisms in soils." Appl Environ Microbiol **75**(7): 2046-2056.

Whittenbury, R., K. C. Phillips and J. F. Wilkinson (1970). "Enrichment, isolation and some properties of methane-utilizing bacteria." J Gen Microbiol **61**(2): 205-218.

Wu, D., J. Raymond, M. Wu, S. Chatterji, Q. Ren, J. E. Graham, D. A. Bryant, F. Robb, A. Colman, L. J. Tallon, J. H. Badger, R. Madupu, N. L. Ward and J. A. Eisen (2009). "Complete genome sequence of the aerobic CO-oxidizing thermophile Thermomicrobium roseum." PLoS One **4**(1): e4207.

References Table S6

Biver, S., and Vandenbol, M. (2013). Characterization of three new carboxylic ester hydrolases isolated by functional screening of a forest soil metagenomic library. *J. Ind. Microbiol. Biotechnol.* 40, 191–200. doi:10.1007/s10295-012-1217-7.

Cheng, X., Wang, X., Qiu, T., Yuan, M., Sun, J., and Gao, J. (2014). Molecular cloning and characterization of a novel cold-adapted family VIII esterase from a biogas slurry metagenomic library. *J. Microbiol. Biotechnol.* 24, 1484–9.

Elend, C., Schmeisser, C., Leggewie, C., Babiak, P., Carballeira, J. D., Steele, H. L., et al. (2006). Isolation and biochemical characterization of two novel metagenome-derived esterases. *Appl. Environ. Microbiol.* 72, 3637–45. doi:10.1128/AEM.72.5.3637-3645.2006.

Jeon, J. H., Kim, S.-J., Lee, H. S., Cha, S.-S., Lee, J. H., Yoon, S.-H., et al. (2011). Novel metagenome-derived carboxylesterase that hydrolyzes β-lactam

antibiotics. *Appl. Environ. Microbiol.* 77, 7830–6. doi:10.1128/AEM.05363-11.

Kim, Y. H., Kwon, E. J., Kim, S. K., Jeong, Y. S., Kim, J., Yun, H. D., et al. (2010). Molecular cloning and characterization of a novel family VIII alkaline esterase from a compost metagenomic library. *Biochem. Biophys. Res. Commun.* 393, 45–9. doi:10.1016/j.bbrc.2010.01.070.

Kim, Y.-O., Park, I.-S., Nam, B.-H., Kim, D.-G., Jee, Y.-J., Lee, S.-J., et al. (2014). A Novel Esterase from Paenibacillus sp. PBS-2 Is a New Member of the. *J. Microbiol. Biotechnol.* 24, 1260–1268.

Mokoena, N., Mathiba, K., Tsekoa, T., Steenkamp, P., and Rashamuse, K. (2013). Functional characterisation of a metagenome derived family VIII esterase with a deacetylation activity on β-lactam antibiotics. *Biochem. Biophys. Res. Commun.* 437, 342–8. doi:10.1016/j.bbrc.2013.06.076.

Nacke, H., Will, C., Herzog, S., Nowka, B., Engelhaupt, M., and Daniel, R. (2011). Identification of novel lipolytic genes and gene families by screening of metagenomic libraries derived from soil samples of the German Biodiversity Exploratories. *FEMS Microbiol. Ecol.* 78, 188–201. doi:10.1111/j.1574-6941.2011.01088.x.

Ouyang, L.-M., Liu, J.-Y., Qiao, M., and Xu, J.-H. (2013). Isolation and biochemical characterization of two novel metagenome-derived esterases. *Appl. Biochem. Biotechnol.* 169, 15–28. doi:10.1007/s12010-012-9949-4.

Petersen, E. I., Valinger, G., Sölkner, B., Stubenrauch, G., and Schwab, H. (2001). A novel esterase from Burkholderia gladioli which shows high deacetylation activity on cephalosporins is related to beta-lactamases and DD-peptidases. *J. Biotechnol.* 89, 11–25.

Rashamuse, K., Magomani, V., Ronneburg, T., and Brady, D. (2009). A novel family VIII carboxylesterase derived from a leachate metagenome library exhibits promiscuous beta-lactamase activity on nitrocefin. *Appl. Microbiol. Biotechnol.* 83, 491–500. doi:10.1007/s00253-009-1895-x.

Schütte, M., and Fetzner, S. (2007). EstA from Arthrobacter nitroguajacolicus Rü61a, a thermo- and solvent-tolerant carboxylesterase related to class C beta-lactamases. *Curr. Microbiol.* 54, 230–6. doi:10.1007/s00284-006-0438-2.

Selvin, J., Kennedy, J., Lejon, D. P. H., Kiran, G. S., and Dobson, A. D. W. (2012). Isolation identification and biochemical characterization of a novel halo-tolerant lipase from the metagenome of the marine sponge Haliclona simulans. *Microb. Cell Fact.* 11, 72. doi:10.1186/1475-2859-11-72.

Takehara, M., Kinoshita, K., Miyamoto, M., and Hirohara, H. (2012). A novel alkaline esterase from Sporosarcina sp. nov. strain eSP04 catalyzing the hydrolysis of a wide variety of aryl-carboxylic acid esters. *Biosci. Biotechnol. Biochem.* 76, 1721–7. doi:10.1271/bbb.120332.

Yu, E. Y., Kwon, M.-A., Lee, M., Oh, J. Y., Choi, J.-E., Lee, J. Y., et al. (2011). Isolation and characterization of cold-active family VIII esterases from an arctic soil metagenome. *Appl. Microbiol. Biotechnol.* 90, 573–81. doi:10.1007/s00253-011-3132-7.
